# Targeted Cancer Immunotherapy: Nanoformulation Engineering and Clinical Translation

**DOI:** 10.1002/advs.202204335

**Published:** 2022-10-18

**Authors:** Meihua Yu, Wei Yang, Wenwen Yue, Yu Chen

**Affiliations:** ^1^ Materdicine Lab School of Life Sciences Shanghai University Shanghai 200444 P. R. China; ^2^ Department of Urology Xinhua Hospital School of Medicine Shanghai Jiaotong University 1665 Kongjiang Road Shanghai 200092 P. R. China; ^3^ Shanghai Engineering Research Center of Ultrasound Diagnosis and Treatment Department of Medical Ultrasound Shanghai Tenth People's Hospital Ultrasound Research and Education Institute Tongji University Cancer Center Tongji University School of Medicine Shanghai 200072 P. R. China

**Keywords:** active targeting, cancer therapy, immune cell, immuno‐drug, nanoformulation

## Abstract

With the rapid growth of advanced nanoengineering strategies, there are great implications for therapeutic immunostimulators formulated in nanomaterials to combat cancer. It is crucial to direct immunostimulators to the right tissue and specific immune cells at the right time, thereby orchestrating the desired, potent, and durable immune response against cancer. The flexibility of nanoformulations in size, topology, softness, and multifunctionality allows precise regulation of nano‐immunological activities for enhanced therapeutic effect. To grasp the modulation of immune response, research efforts are needed to understand the interactions of immune cells at lymph organs and tumor tissues, where the nanoformulations guide the immunostimulators to function on tissue specific subsets of immune cells. In this review, recent advanced nanoformulations targeting specific subset of immune cells, such as dendritic cells (DCs), T cells, monocytes, macrophages, and natural killer (NK) cells are summarized and discussed, and clinical development of nano‐paradigms for targeted cancer immunotherapy is highlighted. Here the focus is on the targeting nanoformulations that can passively or actively target certain immune cells by overcoming the physiobiological barriers, instead of directly injecting into tissues. The opportunities and remaining obstacles for the clinical translation of immune cell targeting nanoformulations in cancer therapy are also discussed.

## Graphical abstract

Cancer immunotherapy aims to direct human immune system to eradicate cancerous cells. Here preclinical and clinical development progress in engineered nanoformulations that direct immune drugs to tissue‐specific subset of immune cells for enhanced cancer immunotherapy is reviewed. The mechanism of action of exemplified nano‐paradigms is particularly highlighted and aggressive exploration of innovative nanoplatforms and nano‐immunology interplay mechanism is encouraged.

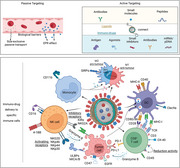

## 1 Introduction

Cancer remains the main worldwide health problem, and diverse therapeutic approaches have been explored over decades. Patients can benefit from chemotherapy, radiotherapy, surgery, and newly developed immunotherapy, relying on the characteristics of tumors. Surgery, if possible, is still the most efficacious approach to clear tumors and associated lymphatics, while patients with advanced cancers need to receive chemotherapy by systemic infusion of cytotoxic anti‐cancer drugs. Nevertheless, side effects associated with off‐target toxicities are often the major limiting factor for the implications and therapeutic outcomes of these cancer therapies.

Nanomaterials typically in the size of 1–200 nm are revolutionizing the development of new medicines, particularly in targeted cancer therapies. Small‐sized nanoformulations tend to passively target solid tumor sites driven by EPR (enhanced permeability and retention) effect, which is caused by the defective angiogenic vessels.^[^ ^1^ ^]^ Therapeutics formulated in nanomaterial delivery platforms were initially developed to promote accumulation of chemotherapeutic agents at tumor sites and reduce unwanted systemic toxicities.^[^ ^2^ ^]^ There are considerable chemotherapeutic nanoformulations licensed for human use, showing substantially improved therapeutic outcomes.^[^ ^3^ ^]^ Emerging advances in nanomedicine have focused on its capability to enhance cancer immunotherapy, by precisely modulating immunosuppressive tumor microenvironment (TME).^[^ ^4^, ^5^, ^6^, ^7^ ^]^

Beginning over 100 years ago, the concept of immune surveillance was proposed by Professor Paul Ehrlich, the winner of 1908 Nobel Prize in Physiology or Medicine. It is defined that the immune system is capable to recognise and eliminate tumor cells, thus preventing tumor progression.^[^ ^8^ ^]^ Therapeutic vaccination is one key strategy to empower the immune system with augmented tumor antigen specific cytotoxic T lymphocytes (CTLs) against tumor.^[^ ^9^ ^]^ However, the therapeutic efficacy is often compromised by suboptimal T cell priming or immunoresistance in TME. The advances in immuno‐oncology have brought new insights into the area of developing novel immunotherapeutic strategies, many of which have shown remarkable success in curing patients with certain types of cancers, such as checkpoint inhibitors targeting PD‐1/PD‐L1 (programmed cell death‐1 or its ligand), or CTLA‐4 (cytotoxic T‐lymphocyte antigen‐4),^[^ ^10^, ^11^ ^]^ and adoptive T cell therapies using engineered CAR‐T (chimeric antigen receptor T) cells.^[^ ^12^, ^13^, ^14^, ^15^ ^]^ These ground‐breaking findings fueled a surge of immunotherapy under clinical or preclinical studies exploring new mono‐ or combined therapy.^[^ ^16^ ^]^ Combination immunotherapies, such as co­treatment with multiple antibodies against CTLA‐4, PD‐1, or PD‐L1, have shown some increase in efficacy, but also elicited substantial increase in toxicity in patients.^[^ ^17^ ^]^ Unsurprisingly, systemic administration of these inhibitors inevitably augments the adverse effects associated with autoimmune toxicities.^[^ ^18^ ^]^ Beyond that, other immuo‐drugs (e.g., cytokines, receptor agonists) that modulate the functions of specific subsets of immune cells, also face the safety issue related to systemic toxicities. Thus, additional approaches that could safely and effectively drive anti‐cancer immune responses remain an important unmet need.

To gain broader success of cancer immunotherapy, immuno‐drug delivery nanoformulations that enable spatial and temporal regulation of immune response, have emerged in parallel with the discoveries of new immunotherapeutic agents, holding a great promise in addressing these limitations. Anti‐tumor immunity is coordinated by diverse immune cells timely and spatially across different tissues, such as T cell priming in the lymphoid organs,^[^ ^19^, ^20^ ^]^ progenitors circulating in blood before differentiating into tissue specific phenotypes of immune cells,^[^ ^21^, ^22^ ^]^ and effector immune cells exerting their killing functions at tumor sites.^[^ ^23^ ^]^ In terms of delivery function, nanoformulations can be engineered with desired properties^[^ ^24^, ^25^, ^26^ ^]^ to load, protect, and guide the therapeutic immuno‐drugs to the specific subsets of immune cells at the right tissue and right time for enhanced coordination of immune response against tumor. Beyond that, accumulated evidence has also shown that nanomaterial itself can offer adjuvant effect modulating the nano‐immunological activities that are determined by their chemophysical properties, such as chemical components,^[^ ^27^ ^]^ size and solubility,^[^ ^28^ ^]^ softness,^[^ ^29^ ^]^ deformability,^[^ ^30^ ^]^ topology,^[^ ^31^, ^32^ ^]^ and chirality.^[^ ^33^ ^]^ These discoveries in structure‐activity relationships and previously unexplored mechanisms associated with the interface of nano‐immunology have been broadening and advancing the implications of highly immunogenic nanoformulations in targeted cancer therapy.

**Figure 1:**
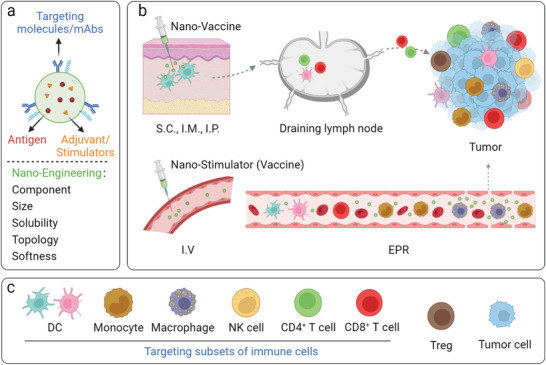
Nanoengineering enhances the immunomodulation of stimulators and expansion of anti‐tumor immunity, by targeting specific type of immune cells. a) Tumor‐associated antigens and/or therapeutic immunostimulatory agents can be formulated in nanoparticles with specific chemophysical properties, including chemical components, particle size, solubility, softness, and topology. b) Chemophysical properties of immunotherapeutic nanoformulations determine the quality of innate and adaptive immune response, by targeting modulation of specific subsets of c) immune cells at lymphatic or tumorous tissues post systemic administration. Created with Biorender.c.

Despite that the tremendous progress in the field of nano‐engineering has enhanced cancer immunotherapy, advanced nanoformulations that enable passive or active targeting delivery of systemically administrated immuno‐drugs to specific subsets immune cells at peripheral, lymphatic, or tumorous tissues, have not yet been timely summarized and clarified. In this review, we mainly focus on the targeting nanoengineered formulations for tissue‐specific immune cell delivery of immuno‐drugs, by passively and actively overcoming physical barriers post systemic administration (**Figure** **1**a,b). We discuss specific nano‐paradigms designed to target DCs, circulating monocytes, macrophages, T cells, or NK cells (Figure 1c) for enhanced cancer therapy. The associated mechanisms are emphasized by which the targeting formulations precisely regulate the anti‐tumor immunity across tissues. We also briefly summarize targeting nanoformulations currently under clinical studies. We conclude this review by providing our perspectives on the potential translations and limitations of immune cell targeting nanoformulation in cancer treatment, such as safety evaluation, establishment of reliable pre‐clinical models, and collaborative research among researchers from material science, engineering, onco‐immunology, bioinformatics, and medicine. This review will provide a comprehensive and up‐to‐date summary of advancements in the exciting multidisciplinary field of material science and onco‐immunology for enhanced cancer therapy.

## 2 Emerging Nanoformulations Targeting Immune Cells for Cancer Therapy

Cancer immunotherapy exploits the extraordinary capability of human immune system to fight against cancerous cells. The increasing knowledge in onco‐immunology has led to innovative cancer invention approaches with several approved products for human use.^[^ ^18^ ^]^ Despite the rapid progress and success in this field, most cancer patients do not response to cancer immunotherapy. It remains a major challenge to develop new modalities to enhance the response rate and therapeutic efficacy while minimize the autoimmune toxicities. The availability of new technologies in nanomaterials enables selective immunomodulation on specific immune cell types or subtypes, which plays a prominent role in tuning the interactions between innate immune cells and adaptive immunity against tumor.^[^ ^34^ ^]^ This approach might offer safe and promising alternative cancer treatment modalities to traditional cancer immunotherapies and therapeutic cancer vaccines. Immune cells possess phenotypic and functional heterogeneity, which are found to function differently in diverse (non)lymphoid tissues. We here will provide a brief introduction to the basic immunological functions of each subset of immune cells in cancer immunotherapy, followed by the remarkable impact of nanoformulations and insight into the key opportunities and issues.

### 2.1 Nanoformulations Targeting DCs

All nucleated tumor cells express major histocompatibility complex (MHC) class I molecules that can present intracellular antigen‐derived peptides on cell surface, thus tumor specific CD8+ CTLs can detect and target tumors. CTLs eliminate cancer cells by producing specific cytokines (e.g., interferon (IFN)‐*γ*) and cytotoxic enzyme molecules (e.g., granzyme).^[^ ^35^ ^]^ Priming CTLs in lymphoid organs by therapeutic vaccination is one preferred tool to establish efficacious anti‐tumor immunity, of which DCs are vital for initiating CTLs.^[^ ^9^, ^36^ ^]^ Targeting DCs therefore is considered as a promising strategy in cancer therapy, such as vaccination with antigens and nano‐immunomodulators that can target and activate endogenous DCs in lymphoid organs. The flexibly engineered nanomaterial‐based immunomodulators that target DCs enable triggering T cell anti‐tumor activity rapidly and effectively.

#### 2.1.1 DCs in Skin Draining Lymph Nodes

Therapeutic vaccine nanoformulations containing small nanomaterials allow for efficient passive targeting transport of adjuvants and antigens to endogenous DCs in draining lymph nodes (dLNs) through afferent lymphatic vessels, thereby promoting presentation of antigens by activated DCs to naïve T cells and subsequently priming antigen specific anti‐tumor effector T cells. Moon lab engineered ≈10 nm disc‐like nanoparticles that were formed by phospholipids and apolipoprotein A1‐mimetic peptides to co‐deliver tumor peptide antigens and adjuvant 5′‐C‐phosphate‐G‐3′ (CpG).^[^ ^37^ ^]^ Following administration, this engineered nanoformulation markedly increased accumulation of delivered antigen and adjuvant molecules in dLNs compared to their free soluble form, and also prolonged the antigen presentation on DCs. Nanoformulation vaccination in mice elicited ≈30‐fold greater CTLs than unpacked antigen and CpG formulation, resulting in enhanced therapeutic efficacy against B16F10 melanoma and MC38 colon cancer. Vaccines made up of physically mixed adjuvants and antigens might induce immune tolerance and reduce anti‐tumor immunity, as free antigens are likely drained to LNs and presented by DCs in the absence of adjuvants that provide positive stimulatory signals.^[^ ^38^ ^]^

**Figure 2:**
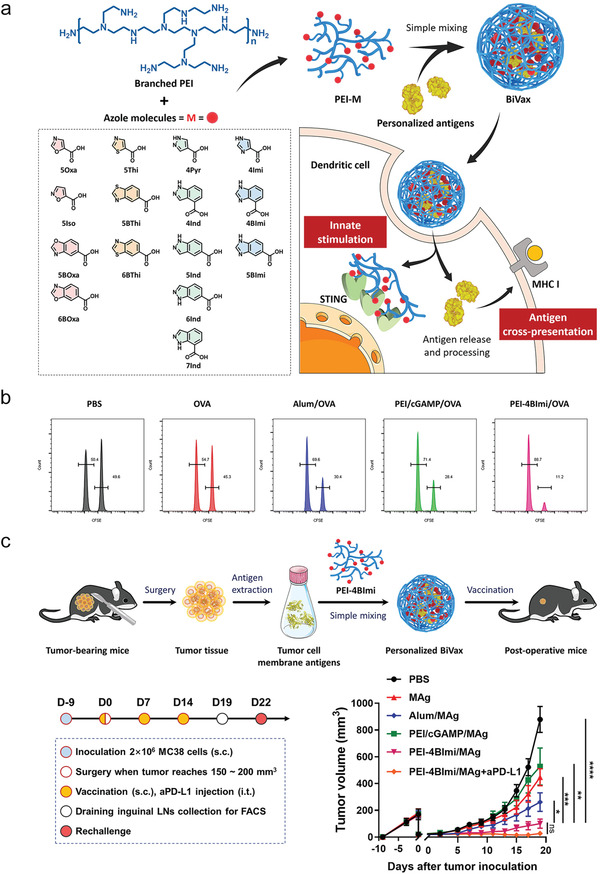
Azole (M) architecture controls the activation of STING pathway and anti‐tumor activity of PEI‐M vaccine nanoformulation. a) Illustrated are the preparation process of PEI‐M encapsulated vaccine nanoformulation, the chemical structures of different Azole molecules, and activation and antigen presentation pathways of innate immune cells. b) Flow cytometry analysis of CTL killing effect in vivo. c) Illustrated are the experimental process and timeline in mice bearing with MC38 tumor. Shown are MC38 tumor growth curves in mice received with different treatments as indicated. a–c) Reproduced with permission.^[^ ^43^ ^]^ Copyright 2022, Wiley‐VCH.

In addition to passive targeting DCs in dLNs, some nanomaterials are engineered to promote cytosolic delivery‐mediated cross presentation of delivered antigens via MHC class I molecules of DCs, which is essential for increasing the magnitude of CD8+ T cell responses.^[^ ^39^ ^]^ Gao and his colleagues reported ultra‐pH‐sensitive polymer nanoparticles with a diameter of ≈30 nm to deliver a model antigen of ovalbumin (OVA) without additional adjuvants.^[^ ^40^ ^]^ The designed architecture of cyclic seven‐membered ring endowed this nanoparticle with potent immunogenicity on DC maturation by activating stimulator of interferon genes (STING)‐type I IFN pathway. Exogenous nanoparticles are generally uptaken by DCs via endocytosis pathway and trapped in acidic endosome compartments.^[^ ^41^ ^]^ However, the tertiary amine group in the cyclic ring facilitated the disruption of endosomes, cytosolic delivery of exogenous OVA, and cross presentation. Following recognition of cross presented antigens, mature DCs stimulated the differentiation and proliferation of OVA‐specific functional effector CTLs. Immunization with the nanoformulation, led to efficacious elimination of B16F10, MC‐38, and human papilloma virus (HPV) associated tumors. This intriguing nanoformulation is projected in first human trials in 2023. The authors recently engineered a polyvalent STING nano‐agonist derived from the polymer system with prolonged activation.^[^ ^42^ ^]^ Very recently, Song and co‐workers screened a library of azole molecules for a potent STING activation structure 4BImi (as illustrated in **Figure** **2**a), which was conjugated with polyethylenimine (PEI) mediating endocytic delivery of encapsulated antigens.^[^ ^43^ ^]^ The designed vaccine nanoformulation (50–100 nm) elicited potent CTLs when OVA was formulated into the polymer nanoparticles (Figure 2b). This strategy also showed a great potential in a personalized vaccine formulation, substantially regressing MC38 tumor (Figure 2c). These innovative nano‐STING agonists in the format of nanoparticles with improved stability in vivo^[^ ^44^ ^]^ represent an attractive approach for cancer therapy in parallel with new discoveries in small molecular STING agonists.^[^ ^45^, ^46^, ^47^, ^48^ ^]^

**Figure 3:**
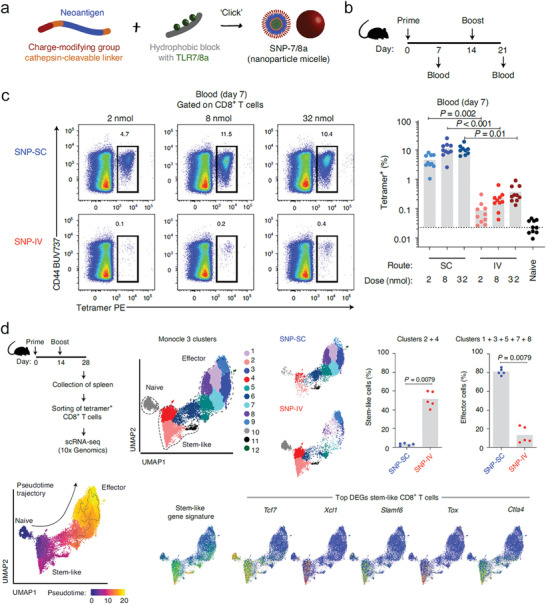
The administration route and dose of TLR‐7/8 agonist‐based vaccine nanoformulation determine the level and phenotype of neoantigen‐specific CD8+ T cells. a) An illustration showing the fabrication procedure of self‐assembling nanoparticle vaccines formed by MC38 neoantigen (Reps1) and TLR7/8 agonist (SNP‐7/8a). b) An experimental timeline: C57BL/6 mice received SNP‐7/8a vaccine (dose of 2, 8, 32 nmol) subcutaneously or intravenously on days 0 and 14. Blood samples were harvested on days 7 and 21 for the measurement of Reps1 specific CD8+ T cells. c) Reps1‐tetramer+ CD44+CD8+ T cell percentages were determined by flow cytometric analysis and displayed in the representative dot plots and bar charts. d) Mice received SNP‐7/8a Reps1 peptide vaccine subcutaneously or intravenously on days 0 and 14 and Reps1‐tetramer+CD8+ T cells were sorted from the spleen on day 28. The sorted cells were used for single cell sequencing (scRNA seq) analysis by 10× Genomics. Monocle 3 analysis of gene expression displayed 12 distinct clusters in the uniform manifold approximation and projection (UMAP) of the sorted cells. Shown are the quantified percentages of effector and stem‐like cells of total sorted cells, Pseudotime trajectory analysis, and typical gene signatures of stem‐like phenotype of CD8+ T cells overlaid on the UMAP. a–d) Reproduced with permission.^[^ ^50^ ^]^ Copyright 2020, Nature Publishing Group.

Seder and colleagues developed a potent peptide vaccine nanoformulation in the absence of additional nanomaterial carriers.^[^ ^49^, ^50^ ^]^ The chemically modified peptides were covalently linked with a molecule adjuvant of toll‐like receptor (TLR)‐7/8 agonist (**Figure** **3**a). The conjugates were capable of self‐assembling into nanoparticles (denoted as SNP‐7/8a, ≈20 nm), which promoted the activation of targeted DCs in dLNs. Mice subcutaneously immunized with the nanoformulation exhibited a high magnitude of tumor specific CD8+ T cells.^[^ ^49^ ^]^ Along with magnitude, the functional quality and longevity of CD8+ T cells are critically important for efficient and durable anti‐tumor immunity. Interestingly, the lab found that the administration route of the nanoformulation vaccine dramatically altered the targeted DC subtype, antigen presentation persistence, phenotype of functional CD8+ T cells, and therapeutic outcomes against cancer.^[^ ^50^ ^]^ By comparing to subcutaneous vaccination, intravenous vaccination with SNP‐7/8a nanoformulation resulted in a substantially lower magnitude of neoantigen+CD8+ T cells in all tested tissues (blood (Figure 3b,c), spleen, LNs, and lungs), but higher proportion of stem‐like phonotype expressing T cell factor 1 (TCF1) and PD‐1 markers (Figure 3d) that demonstrated effective control on established MC38 tumor in a therapeutic model. Intravenous administration of vaccine nanoformulation targeted in splenic conventional type 1 DCs (cDC1s) and monocyte‐derived DCs (mDCs) and the delivered antigens were of short‐lived pharmacokinetics (peaked at 6 h and diminished at 24 h). In contrast, subcutaneous administration led to a long duration of antigens in dLN‐cDC1s and prolonged DC activation up to 3 days, which probably accounted for the high number of effector CD8+ T cells. The frequency of cDC1s that are the major subset of DCs for antigen cross‐presentation to CD8+ T cells, was significantly reduced post injection of SNP‐7/8a nanoformulation intravenously, thus the authors proposed that monocyte‐derived DCs might be the DC subset mediating stem‐like CD8+ T cells, while this hypothesis needs to be validated in future evaluation. Chen and colleagues also formulated a therapeutic tumor vaccine by self‐assembling dual adjuvants (CpG and short hairpin RNA) and neoantigens into nanocapsules (around 250 nm) potently activating DCs in dLNs and subsequently priming CD8+ T cell immunity.^[^ ^51^ ^]^ These findings from peptide‐based vaccine nanoformulation provide implications in rational design of effective therapeutic vaccines for cancer treatment.

MHC class I restricted peptide‐based vaccines are suitable for personalized cancer treatment, while unlikely applied in patients universally due to polymorphic human leukocyte antigen (HLA) molecules.^[^ ^52^ ^]^ In contrast, therapeutic messenger RNA (mRNA) cancer vaccines that can deliver whole antigen proteins are able to provide a variety of HLA epitopes. Anderson lab screened a library of ionizable lipid nanoparticles (LNPs, 100 nm in diameter) to optimize mRNA vaccination performance.^[^ ^53^ ^]^ Like the polymer‐based nanoformulation mentioned above, the cyclic amino head groups in LNPs were found capable of activating STING‐type I IFN pathway, thereby significantly promoting the immunogenicity of mRNA vaccine nanoformulation and inducing APC maturation in dLNs. This nanoformulation triggered anti‐tumor immunity and demonstrated efficacious therapeutic effects in multiple mouse tumor models. The developed LNPs were protected in a filed patent for future potential clinical use.

In addition to passive targeting driven by small particle sizes less than 100 nm, active targeting strategies offer an alternative solution to augment tumor antigen specific immunity against tumor, by conjugating ligands or monoclonal antibodies (mAbs) on the surface of nanomaterials. Dolcetti and his colleague Thomas reported an oil‐in‐water nanoemulsion system (200 nm) conjugated with targeting mAb to Clec9a expressed by cross presenting cDC1s.^[^ ^54^ ^]^ By encapsulating tumor antigens, the nanoemulsion vaccine formulation in the absence of additional adjuvants was able to target and activate cross‐presenting Clec9a+DCs, thereby enhancing induction of antigen specific T cells. Immunization with the nanoemulsion vaccine formulation containing HPV E6/E7 protein or pooled B16F10 melanoma neoepitopes, showed significant suppression on the growth of HPV16 expressing TC‐1 solid tumor and B16F10 tumor, respectively. Not only cross priming CD8+ T cells, but Clec9a+cDC1s are also capable of presenting glycolipid on CD1d, MHC class I like molecules, to activate invariant natural killer T (iNKT). The activated iNKT cells in turn provide helper signals to promote DC maturation and consequent antigen‐specific T cell immunity. Taking consideration of this point, Dolcetti lab encapsulated E7 peptide and glycolipid ligand *α*‐galactosylceramide (*α*GC) in the Clec9a‐targeted nanoemulsion, to explore the feasibility of enhanced anti‐tumor immunity driven by NKT cells.^[^ ^55^ ^]^ A single intravenous administration of the designed vaccine nanoformulation enhanced the activation of cDC1s, iNKT cells, NKT cells, and functional effector CD8+ T cells, resulting in long‐term TC‐1 tumor suppression in a therapeutic model. Recently, the Clec9a targeted nanoemulsion vaccine formulation containing whole OVA protein antigen showed improved immunotherapy efficacy of CAR‐T cells that express transgenic T cell receptors (TCRs) specific to MHC class I or II restricted peptides.^[^ ^55^ ^]^ Transgenic C57BL/6‐HER2 (human epidermal growth factor receptor 2) mice received the combined treatments completely regressed E0771‐Her2 breast cancer and MC38‐HER2 tumors.

**Figure 4:**
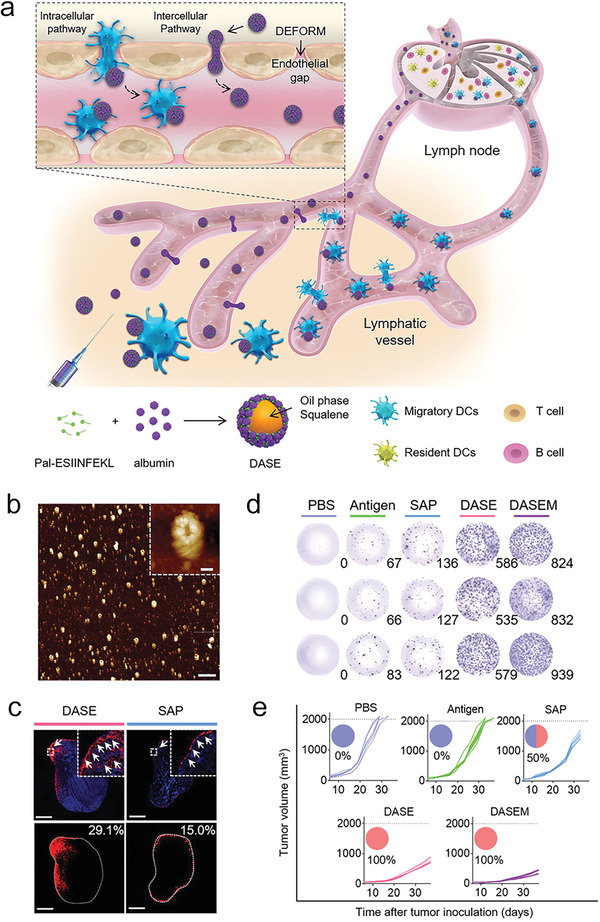
The deformability of vaccine nanoformulation controls its drainage to LNs, the magnitude of antigen specific CD8+ T cells and therapeutic effect against tumor. a) A scheme illustrating the pathway of deformable vaccine nanoformulation draining to the LNs by adjusting its morphology to pass through the endothelial barriers of lymphatic vessels. b) Atomic force microscopy images of DASE in a liquid solution. Scale bar: 2 µm; 100 nm (inset). c) Fluorescent images of Cy5.5 conjugated antigens in LNs harvested from mice at 24 h post administration of different vaccine nanoformulations. d) IFN‐*γ* ELISpot images of splenocytes derived from immunized mice. e) Individual E.G7/OVA tumor growth curves of mice received different vaccine formulations as indicated. a–e) Reproduced with permission.^[^ ^30^ ^]^ Copyright 2021, Wiley‐VCH.

Nanovaccine formulations with a relatively large size (above 100 nm) generally are retained at the injection sites.^[^ ^56^, ^57^ ^]^ Interestingly, Ma lab engineered deformable vaccine nanoemulsions (named DASE, **Figure** **4**a) with a large size of ≈330 nm (Figure 4b), which were able to passively target dLNs by deforming their morphology to pass through endothelial gaps and lymphatic vessels (Figure 4c).^[^ ^30^ ^]^ In this vaccine formulation, albumin was applied to stabilize the squalene oil nanodrops, delivering MHC class I restricted OVA peptide SIINFEKL conjugated with a lipid chain palmitic acid (Pal‐ESIINFEKL).^[^ ^30^ ^]^ The deformable vaccine nanoformulation induced significantly enhanced SIINFEKL‐specific CD8+ T cell immune response (Figure 4d), thereby promoting therapeutic effects against OVA expressing E.G7‐OVA thymoma (Figure 4e), compared to albumin‐based solid counterparts. Further preclinical studies on the delivery of whole tumor‐associated protein antigens would pave a path to the use of deformable nanoformulation in therapeutic vaccination.

The effect of nanoparticle chirality on the regulation of nano‐immunological activities had been unknown until early this year. Xu and colleagues discovered that left‐handed inorganic gold nanoparticles exhibited substantially stronger immune response against infectious disease^[^ ^33^ ^]^ and cancer,^[^ ^58^ ^]^ compared to the right‐hand counterparts. However, the interaction between chiral nanoparticle‐formed cancer vaccine and DCs in vivo was not explored but might be interesting to fully understand the underlying mechanisms.

**Figure 5:**
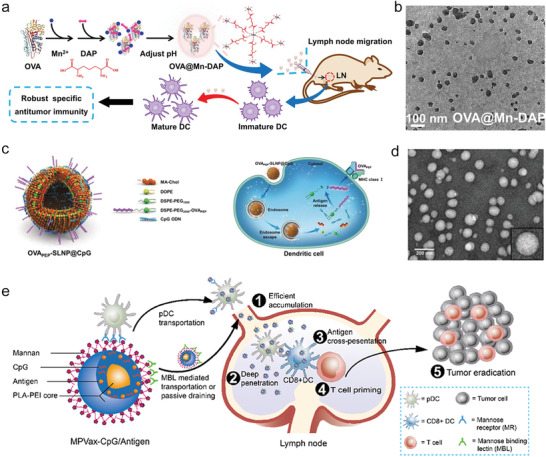
Vaccine nanoformulations coupled with agonists activate DC receptor signaling pathway to promote anti‐tumor immunity. Illustrated are the construction and mode of actions of a) OVA/diaminopimelic acid/Mn^2+^/Nod1 agonist, c) OVA/cationic cholesterol LNP/CpG, and e) polylactic acid/PEI/CpG/mannan nanoformulations on dLN‐DCs for improved anti‐tumor activity. Transmission electron microscopy (TEM) images of b) OVA/diaminopimelic acid/Mn^2+^/Nod1 agonist and d) OVA/cationic cholesterol LNP/CpG. a,b) Reproduced with permission.^[^ ^62^ ^]^ Copyright 2019, American Chemical Society. c,d) Reproduced with permission.^[^ ^63^ ^]^ Copyright 2020, Wiley‐VCH. e) Reproduced with permission.^[^ ^64^ ^]^ Copyright 2022, Elsevier.

Several studies have reported that therapeutic outcomes in cancer treatment can be substantially improved by specifically activating other dLN‐DC receptor signaling using innovative small nanomaterials,^[^ ^59^, ^60^, ^61^, ^62^, ^63^, ^64^ ^]^ such as polymer/metal ions nanoparticles (**Figure** **5**a,b),^[^ ^62^ ^]^ cationic cholesterol LNPs (Figure 5c,d),^[^ ^63^ ^]^ and pathogen‐like polymer nanoparticles (Figure 5e).^[^ ^64^ ^]^ Harnessing the potential of targeting dLN‐DCs as a deliberate strategy to trigger CTL‐mediated cancer eradication is far beyond what can be achieved by the representative fascinating examples introduced above. This paradigm is expected to deliver more possibilities for advancing targeted cancer therapies.

#### 2.1.2 DCs at Tumor Sites

As opposed to therapeutic vaccination in the periphery with exogenously derived and well‐defined tumor‐associated antigens (TAAs) or tumor specific antigens (TSAs), in situ vaccination (ISV) in TME sourced the endogenous antigens from the dying cancer cells, which could be presented to tumor infiltrating DCs to generate adaptive T cell immune response.^[^ ^36^, ^65^, ^66^ ^]^ ISV approach completely avoids the challenges in some tumors where specific antigens are hard to be defined. This strategy also provides entire gamut epitopes that are unique to each individual. ISV approach was first practiced by a cancer surgeon Coley who observed that surgical site infection occasionally happened in some patients led to shrinkage of unremoved tumors, and then purposely injected bacterials into patients for cancer treatment with a fraction of responders,^[^ ^67^ ^]^ though the concept of ISV was not defined or realized yet. Various ISV methods that aim to induce immunogenic cell death (ICD) or activate DCs are currently under clinical studies, such as agonists of TLR, STING and CD40 receptor, Fms‐like tyrosine kinase 3 ligand (FLT3L), oncolytic viruses, and radiotherapy. Monotherapies and combined therapies of these ISV candidates under clinical consideration are introduced in detail elsewhere.^[^ ^9^ ^]^ We here in this section will focus on recent advanced nanoformulations targeting DCs in TME for enhanced ISV efficacy.

Taking advantage of passive tumor accumulation and capability of delivering multiple ISV agents, our lab engineered a liposome‐based nanoformulation (≈160 nm) co‐encapsulating a sonosensitizer hematoporphyrin monomethyl ether (HMME) and a TLR7 agonist (R837).^[^ ^68^ ^]^ Ultrasound responsive HMME enhanced sonodynamic therapy (SDT)‐induced ICD, which in turn promoted DC activation in the combined immunostimulator R837, antigen presentation, and T cell priming. The enhanced ISV in combination with anti‐PD‐L1 mAbs significantly improved therapeutic effect against 4T1 and CT26 tumors. Recently, Wang lab reported a functional polymer‐based nanoformulation (named as SPNI, with a diameter of 35 nm) consisting of semiconducting polymer for near‐infrared (NIR) light‐mediated photodynamic therapy (PDT) and amphipathic polymer for delivery R837 via an acid‐labile linker.^[^ ^69^ ^]^ Upon reaching the tumor site, R837 was released in response to acidic TME, activating DCs infiltrated in tumor. Under NIR light irradiation, SPIN induced reactive oxygen species (ROS)‐mediated ICD, promoting antigen presentation on mature DCs and priming CD8+ T cells against a metastatic 4T1 tumor.

Biomimetic nanoformulations are an increasingly prevalent technology in vaccine design.^[^ ^70^ ^]^ The decoration of nanoformulations with cell membrane enables targeted delivery mediated by natural ligands and immunological modulation. The cell membranes with diverse functions can be prepared from a wide range of cell types, such as red blood cells, tumor cells, and leukocytes. Coating nanoformulations with erythrocyte membranes could prolong circulation time by alleviating clearance by immune cells.^[^ ^71^ ^]^ Leukocyte membrane triggers the activation of anti‐tumor immunity of nanoformulations.^[^ ^72^ ^]^ Nanoformulations decorated with tumor cell membranes allow their homologous targeting to tumor sites.^[^ ^73^ ^]^ In the context of targeted cancer immunotherapy, Gao and his colleagues engineered PLGA (poly(lactic*‐co‐*glycolic acid)) nanoparticles (≈150 nm in diameter) coated with biomimetic cancer cell membrane expressing peptides that can specifically target Clec9a expressed on the surface of cDC1s.^[^ ^74^ ^]^ The developed nanoplatform enabled active targeting delivery of co‐encapsulated 2′3′‐cGAMP (2'3'‐cyclic‐guanosine monophosphate‐adenosine monophosphate, a STING agonist) and tumor antigen to Clec9a+DCs, stimulating type I IFN secretion, DC maturation, and cross presentation of antigens. Immunization with the vaccine nanoformulation promoted antigen specific polyfunctional CD8+ T cell immune response, consequently contributing advanced therapeutic effect against B16F10‐OVA and 4T1 tumors, particularly in combination with radiotherapy.

There are considerable evidence and great implications for the use of therapeutic nanoformulation vaccination in cancer immunotherapy, by targeting DCs in the periphery or TME, which largely determine the initiation and shaping of adaptive immune response against tumor. The benefits obtained from these nanoformulations in cancer therapy can be contributed to 1) the tailored nanoscale sizes, and flexible manipulation of surface properties and elasticity for enhanced LN drainage, 2) prevention of tolerogenic effects by co‐delivering of immune stimulators and antigens, and avoiding free antigens being taken up by immature or semi‐mature DCs; 3) prolonged antigen retention in DCs, to enhance antigen presentation efficacy and T cell priming capability for long‐term and polyfunctional antitumor immunity. In the context of tumor‐DCs, the insights into the tumor‐derived factors that impair the functions of DCs in vivo through multiple mechanisms,^[^ ^75^, ^76^, ^77^, ^78^ ^]^ will provide guidelines for the development of next generations of nano‐strategies targeting tumor specific DCs.

### 2.2 Nanoformulations Targeting T Cells

Therapeutic cancer vaccine effectiveness is often compromised by T cell exhaustion that can be driven by the suboptimal priming of CTLs (such as persistent antigenic stimulation, lack help signals from DCs and CD4+ T cells)^[^ ^79^, ^80^ ^]^ or the existence of inhibitory signals from tumor or stromal cells.^[^ ^81^ ^]^ Exhausted T cells display a dysfunctional state, losing their capability of producing cytokines and cytotoxic enzymes against tumor cells. Functionally impaired T cells are heterogeneous, with increased expression of inhibitory receptors of PD‐1, lymphocyte activation gene 3 (LAG3), T cell immunoglobulin and mucin domain 3 (TIM‐3), eomesodermin (EOMES), CTLA‐4, and/or CD244. Immunotherapies by a blockade of the inhibitory receptor signaling are an effective strategy in cancer treatment, though only a small fraction of patients (≈20%) can benefit from this approach.^[^ ^81^ ^]^

**Figure 6:**
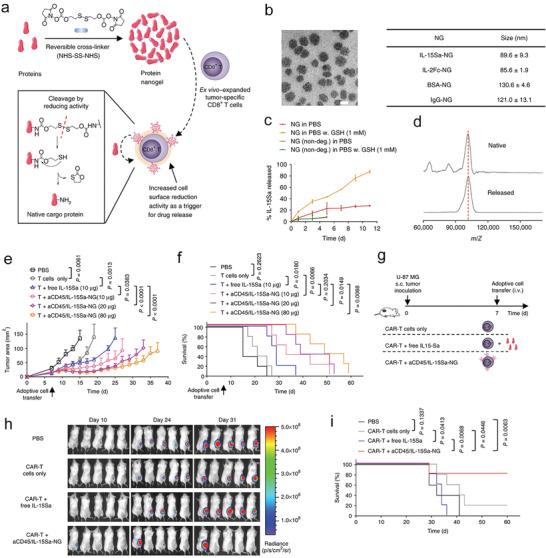
Adjuvant drugs formulated in protein nanogels substantially improve therapeutic effect of T cell therapy, by specifically activating T cells in a TCR‐signaling responsive manner. a) A scheme illustrating the formation of protein nanogels (NG) and its triggered release of adjuvant drugs by the reduction activity of primed CD8+ T cells. b) A TEM image of IL‐15Sa‐NG and the hydrodynamic sizes of IL‐15Sa‐NG, IL‐2Fc‐NG, BSA‐NG, and IgG‐NG measured by dynamic light scattering. Scale bar: 50 nm. c) The release profiles of IL‐15Sa in the forms of redox‐responsive or non‐degradable NG. d) Free or released cytokines measured by a matrix assisted laser desorption/ionization (MALDI) mass spectrometry. e) The average B16F10 tumor growth curves and f) survival rates of mice administrated intravenously with different formulations as indicated. g) Experimental timeline: NSG mice were inoculated subcutaneously with luciferase‐U‐87 MG cells on day 0, followed by intravenous transfer of adoptive human T cells that contained CAR‐T cells targeting epidermal growth factor receptor (EGFR) on day 7. Mice received different treatments as indicated. U‐87 MG tumors in mice received with different treatments were detected by bioluminescence in vivo imaging at h) different time points and i) the survival curves are shown. a–i) Reproduced with permission.^[^ ^85^ ^]^ Copyright 2018, Nature Publishing Group.

Adoptive transfer of tumor‐specific T cells that are engineered or expanded ex vivo is an alternative curative therapeutic option, which has demonstrated extraordinary success in the treatment of patients with leukemia or melanoma.^[^ ^82^, ^83^ ^]^ Nevertheless, it remains of great interest to enhance T cell therapy with increased portion of responders and extended therapeutic effects to other types of solid tumors. The administration of TME‐immunomodulators or cytokines is a central approach under preclinical and clinical studies for enhanced T cell therapy, while these strategies usually show substantial toxicity in patients due to off‐target effects. Rationally engineered nanoparticles that are conjugated with or encapsulated immunodrugs enable active targeting to the surface of T cells and subsequent modulation in a controlled manner. Schmid et al. reported PLGA polymer decorated with polyethylene glycol (PEG) and anti‐CD8a antibodies, capable of targeting CD8+ T cells that were circulating in mouse blood, or resident in lymphoid organs and tumors.^[^ ^84^ ^]^ To target a phenotype of exhausted CD8+ T cells in tumor, anti‐PD‐1 antibody was conjugated on the surface of PLGA/PEG nanoparticles. The delivery of a transforming growth factor‐beta receptor 1 (TGF*β*R1) inhibitor (SD208) that obstructed TGF*β* signaling mediated immunosuppression, enabled restoring the function of effector CD8+ T cells against MC38 tumor, while free drugs showed limited therapeutic effect. This targeting strategy was also able to deliver a TLR7/8 agonist (R848) of innate immunity, increasing the response of MC38 and B16F10 tumors to anti‐PD‐1 mAbs. In contrast, the combination of free R848 and anti‐PD‐1 mAbs had no effect. Sustained release of R848 and specific delivery enabled significantly improved infiltration of CD8+ T cells, accounting for the superior therapeutic effect in cancer treatment. Inspired by the fact that primed T cells possess a higher level of reductive thiols on the cell surface than T cells with a naive phenotype (**Figure** **6**a), the same lab reported reduction‐responsive protein nanogels (80–130 nm) that were formed by cross linking the protein molecules (interleukin (IL)15Sa, IL‐2Fc, bovine serum albumin (BSA) or IgG) using NHS‐SS‐NHS (Figure 6b).^[^ ^85^ ^]^ The disulfide groups allowed responsive release of cargo proteins in the presence of reducing agents of glutathione (GSH) (Figure 6c,d) or activated T cells stimulated with anti‐CD3/CD28 antibodies. Surface conjugation of nanogels with anti‐CD45 specific antibodies prolonged the retention of nanogels specifically on the surface of naïve T cells up to 7 days, preventing fast cell internalization of carried IL15Sa. Systemic administration of IL15Sa nanogels together with adoptive transferred mouse T cells or human CAR‐T cells substantially improved therapeutic effect against B16F10 tumor (Figure 6e,f) or U‐87 MG human glioblastoma (Figure 6g–i) in vivo, compared to systemic administration of free IL15Sa. Chen lab designed a multifunctional nanoformulation (≈145 nm), which comprised 1) magnetic iron oxide nanoparticles for tumor accumulation under an external magnetic field; 2) fucoidan with inherent therapeutic effect and immunostimulatory functions; 3) aldehyde‐dextran for conjugation of anti‐PD‐L1 mAbs (checkpoint inhibition) and anti‐CD3/CD28 mAbs (T cell activation).^[^ ^86^ ^]^ Post intravenous administration, this multiple functional nanoformulation augmented effector T cell mediated anti‐tumor and anti‐metastatic capabilities in 4T1 and CT‐26 tumor models.

Agonistic OX‐40 antibodies currently under clinical studies are effective in stimulating T cell activation and proliferation for enhanced anti‐tumor activity,^[^ ^87^ ^]^ while the corresponding stimulatory receptor OX‐40 molecules can be downregulated on T cells in TME. To promote OX‐40 stimulatory signaling, Dong lab screened a library of phospholipid nanoparticles to optimize the delivery efficacy of OX‐40 mRNA in vitro and in vivo, thereby increasing OX‐40 receptor expression levels on T cells in TME.^[^ ^88^ ^]^ This T cell targeting strategy significantly enhanced therapeutic effect of anti‐OX‐40 antibodies in A20 and B16F10 tumor models, particularly in combined treatments with anti‐CTLA‐4 and anti‐PD‐1 mAbs. Stephan and colleagues designed a stable and effective polymer‐based nanoformulation with a size of ≈155 nm to achieve T cell targeting delivery of a plasmid DNA encoding tumor specific CARs in vivo, instead of engineering CAR‐T cells ex vivo.^[^ ^89^ ^]^ Specifically, the biodegradable poly (*β*‐amino ester) (PbAE) polymer nanoformulation was functionalized with 1) anti‐CD3 antibodies for targeting circulating lymphocytes; 2) microtubule‐associated and nuclear localization signal peptides for targeting the cell nuclear via microtubule transport machinery. The multifunctional nanoformulation enabled selective targeting delivery of DNA molecules to T cells persistently expressing leukemia‐specific 194‐1BBz CAR, leading to potent regression of Eµ‐ALL01 leukemia.

**Figure 7:**
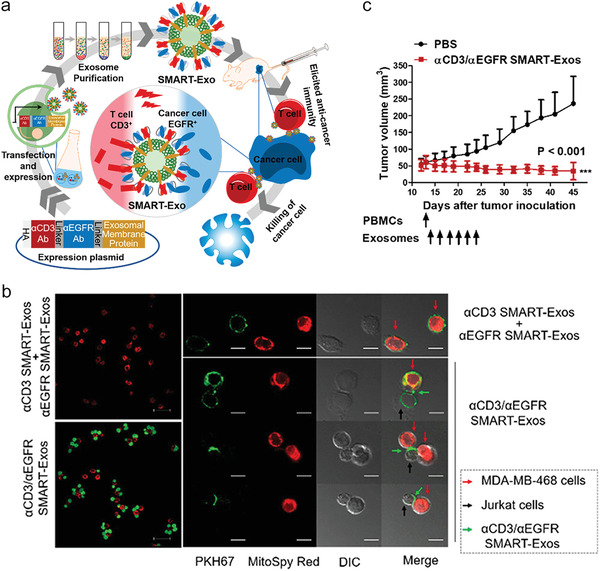
Exosomes genetically engineered with divalent mAbs specially targeting CD3+ T cells and EGFR+ tumor augment T‐cell mediated anti‐tumor immunity. a) A scheme illustrating the preparation of *α*CD3 and *α*EGFR expressing exosomes (defined as SMART‐Exos). b) Confocal images of Jurkat T cells and MDA‐MB‐468 cells (red) linked by SMART‐Exos (green). c) Immunodeficient NSG mice were inoculated with MDA‐MB‐468 cells subcutaneously, followed by intraperitoneal injection of human PBMCs. The next day, mice were intravenously received *α*CD3/*α*EGFR SMART‐Exos or PBS for six times with an interval of 2 days. a–c) Reproduced with permission.^[^ ^93^ ^]^ Copyright 2018, American Chemical Society.

**Figure 8:**
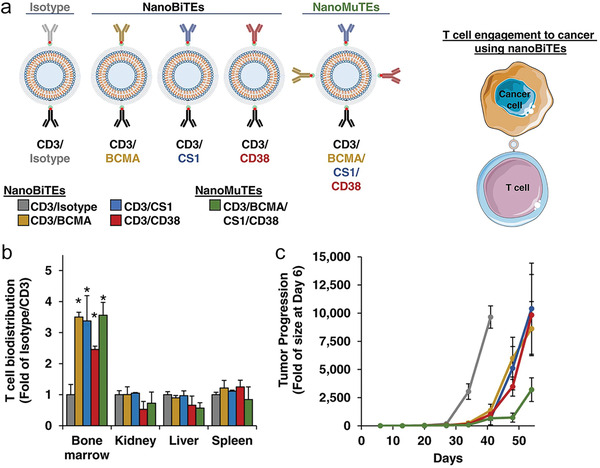
Multiple specific T cell nano‐engagers increase the half‐life of therapeutic mAbs and prevent tumor escape caused by antigen loss, conferring improved cancer immunotherapy. a) Illustrated are engineered different T cell nanoengagers as indicated and bispecific T cell nanoengagers‐mediated tumor killing. b) The biodistributions of adoptive human T cells and c) multiple myeloma progression curves in NSG mice received with different nanoengagers as indicated in (a). a–c) Reproduced with permission.^[^ ^94^ ^]^ Copyright 2021, Nature Publishing Group.

Different from exogenous synthetic nanocarriers, endogenous exosomes are natural membrane vesicles secreted by cells,^[^ ^90^, ^91^, ^92^ ^]^ showing low immunogenicity and reduced clearance possibility by circulating immune cells (such as monocytes and macrophages). Zhang lab engineered an interesting exosome‐based nanoformulation named SMART‐Exos (synthetic multivalent antibodies retargeted exosomes, **Figure** **7**a),^[^ ^93^ ^]^ which were derived from genetically edited HEK293 cells expressing anti EGFR and anti‐CD3 mAbs to link T cells and EGFR‐expressing MDA‐MB‐468 human breast cancer cells (Figure 7b). Following intravenous administration, *α*CD3/*α*EGFR SMART‐Exos were able to target (by *α*CD3) and then direct (by *α*EGFR) the T cells of transplanted human blood mononuclear cells (PBMCs) to MDA‐MB‐468 breast tumor in immunodeficient NSG mice, eliciting potent anti‐tumor immunity (Figure 7c). Recently, Azab and colleagues developed decorated LNPs with multiple mAbs against T cell receptors and tumor antigens, enhancing the engagement between T cell and tumor cells (**Figure** **8**a).^[^ ^94^ ^]^ The designed T cell multiple specific nanoenagers exhibited substantially increased half‐life and T cell accumulation at the tumor site (bone borrow, Figure 8b), compared to iso‐ and bispecific‐nanoenagers. The superior anti‐tumor activity of the multiple specific nanoenagers was demonstrated in an aggressive multiple myeloma mouse model (Figure 8c).

Nanoformulations enabled responsive production of immunomodulators under external stimulus (e.g., lights and ultrasound), thereby offering a strategy to enhance anti‐tumor efficacy of adoptive T cells infiltrated in TME in a controlled manner. Recently, Kwong laboratory utilized PEG‐coated gold nanorods (13 × 47 nm) to passively target tumor infiltrating engineered T cells.^[^ ^95^ ^]^ Under NIR light irradiation at tumor site, gold nanorods mediated thermal control on the expression of IL15Sa or NKG2D stimulatory receptors by engineered adoptive *α*CD19 CAR T or Pmel‐1 specific T cells, significantly improving therapeutic outcomes in multiple tumor models (K562, MDA‐MB‐468, and B16F10). The localized photothermal control strategy would enable targeted delivery of other immuno‐drugs to augment T cell potency against tumor and reduce off‐target toxicity. However, the penetration depth of NIR light is limited (a few centimeters), which greatly hinders the applicability of these strategies in clinic. Therefore, a plasmonic transducer is most likely required in practical applications. Alternative heating modalities, such as ultrasound, will address the limitations and have showed a great potential in thermal control of T cell gene expression.^[^ ^96^ ^]^ This concept provides localized modulation of adoptive T cells by external stimuli for improved efficacy in the treatment of solid malignancies.

Regulatory T cells (Tregs) are a crucial subset of CD4^+^ T cells expressing FOXP3, which mediate immune tolerance by suppressing immune response against self‐antigens as well as tumor antigens.^[^ ^97^ ^]^ In the context of TME a relatively high ratio of Tregs to effector T cells suppresses anti‐tumor immunity, promoting tumor progression. Accumulating evidence has demonstrated that cancer immunotherapy with therapeutics depleting Tregs can effectively enhance anti‐tumor activity.^[^ ^97^ ^]^ Nevertheless, depletion of Tregs is also able to elicit deleterious autoimmunity at non‐tumor tissues. Kim and co‐workers developed neuropilin‐1 (Nrp1) decorated PLGA/lipid hybrid nanoparticles for targeted delivery of anti‐CTLA‐4 mAbs to Nrp1 receptor expressing intratumoral Tregs.^[^ ^98^ ^]^ The engineered nanoformulation elevated the frequency of tumor infiltrating CD8^+^ T cells by blocking Tregs‐mediated immunosuppression, thereby enhancing tumor regression.

T‐cell‐based therapy has transformative potential as alternative modalities in cancer treatment. The scientific breakthroughs have led to three approved products in the clinic. There is a large pipeline of investigative T cell therapies with hundreds of clinical trials underway. Despite the advancements of T cell therapies, innovative strategies are necessary to fulfill their potential in cancer treatment, such as 1) promoting T cell activation and proliferation in vivo; 2) preventing T cell exhaustion and off‐tumor target effects; 3) redirecting efficient T cells tracking to the tumor site. Nanomaterial‐based targeting formulations exemplified above have shown their unique capabilities to meet these optimization criteria. Beyond targeting and modulating T cells in vivo, nanoformulations carrying immuno‐stimulators or cytokines might simultaneously stimulate other immune cells, and restore their functions impaired by the immunosuppressive tumor environment, such as DCs, macrophages, NK cells, and monocytes. The unique chemicals or metal ions released from the nanoformulations might also tune the crosstalk between immune cells and stromal cells via activating unexplored signaling pathways. In all, nanoformulations targeting T cells open the possibility of ultimately expanding the T cell therapies to the treatment of solid tumors.

### 2.3 Nanoformulations Targeting Tumor Associated Macrophages and Monocytes

Along with exhausted CD8+ T cells, tumor associated macrophages (TAMs) are predominant immunosuppressive cell types in TME, which play a critical role in tumor progression to malignancy. The frequency of TAMs is often correlated with the resistance to cancer immunotherapies. Therefore, TAMs are an appealing population to target. For example, the use of antibodies to deplete a specific subtype of TAMs,^[^ ^99^ ^]^ or target a scavenger receptor on TAMs^[^ ^100^ ^]^ in vivo was considered as a promising strategy in promoting cancer regression. TAMs are highly heterogeneous with diverse functions among different tumor types, such as promoting tumor migration and invasion, suppressing effector T cell‐mediated anti‐tumor immunity, and stimulating angiogenesis.^[^ ^101^ ^]^ The inherent plasticity of TAMs fosters the polarization from an M2‐like to an M1‐like phenotype. In most types of tumors, immuno‐suppressive M2‐like cells prevail, thus administration of immuno‐stimulatory molecules in TME can reeducate M2‐like cells to M1‐like cells, promoting the therapeutic efficacy of cancer immunotherapies. For instance, IL‐12, agonists of TLRs and CD40, and IFN‐*γ* were found capable of reprogramming TAMs from M2 to M1 phenotype.^[^ ^102^, ^103^, ^104^ ^]^ The use of nanoformulations enabled in vivo targeting delivery of immuno‐drugs to M2 phenotype of TAMs with improved phenotypic conversion toward M1 against cancer.

**Figure 9:**
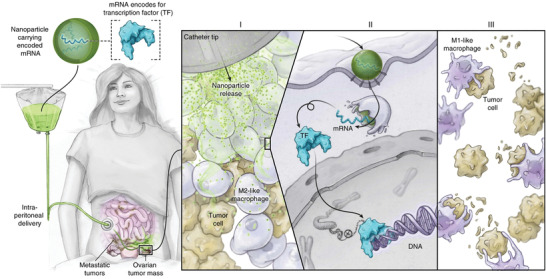
Cationic PbAE polymer nanoformulation delivers anionic mRNA encoding transcription factors (IRF5 and IKK*β*) to ovarian tumor, specifically regulating M1 polarization. Illustrated is the design of planned first clinical trial, in which ovarian cancer patients will receive repeated intraperitoneal infusions of the developed mRNA nanoformulation programing TAMs for therapeutic evaluation. Reproduced with permission.^[^ ^31^ ^]^ Copyright 2019, Nature Publishing Group.

Weissleder lab reported a *β*‐cyclodextrin‐based nanoformulation to encapsulate and deliver R848 molecules to TAMs in vivo, driving M2 to M1 phenotype alternation.^[^ ^105^ ^]^ Mice intravenously administrated with the nanoformulation showed significant regression of MC38 and B16F10 tumors. The combination with anti‐PD‐1 mAbs demonstrated improved immunotherapy outcomes. Of note, R848 delivered by nanoformulations that were discussed above in the section of targeting DCs, was found capable of promoting the maturation of tumor infiltrating DCs, thus it is possible that these R848‐nanoformulations passively targeting to TME might modulate both DCs and TAMs. Instead of delivering immuno‐stimulators, Stephan and colleagues developed cationic  PbAE polymer nanoparticles (≈100 nm) to deliver anionic mRNA encoding interferon regulatory factor 5 (IRF5) and IKK*β*, which were transcription factors regulating M1 polarization in TME.^[^ ^31^ ^]^ IRF5/IKK*β* mRNA‐PbAE nanoformulation demonstrated effective genetic reprogramming macrophages toward M1‐like cells in vivo. Intraperitoneal administration of IRF5/IKK*β* mRNA‐PbAE nanoformulation increased T cell infiltration into solid ID8 ovarian cancer, thereby substantially reducing tumor growth. Following intravenous injection, IRF5/IKK*β* mRNA‐PbAE nanoformulation enabled control on lung metastases in B16F10 model. This approach also improved therapeutic efficacy of radiotherapy in the treatment of glioma. Importantly, repeated intravenous injections of the nanoformulation were found well tolerated without obvious systemic toxicities. This safe and advanced technology is projected to be evaluated in patients with ovarian cancer, as illustrated in **Figure** **9**.

PI3‐kinase *γ* (PI3K*γ*) signaling obstructs the activation of nuclear factor kappa B (NF‐*κ*B) activation via Akt and mammalian target of rapamycin (mTor), which promotes suppressive immune environment during tumor growth. Selective blocking macrophage PI3K*γ* signaling pathway was discovered as a potent strategy in regulating TAM polarization for cancer immunotherapy.^[^ ^106^ ^]^ To achieve long‐term and systemic modulation of immunosuppressive microenvironment, Song et al. recently designed an albumin nanoparticle containing PI3K*γ* inhibitor (IPI‐549) and paclitaxel (PTX), which remodeled the TAM in both tumor‐dLNs and tumor sites via the repolarization of M2 to M1 phenotype, thereby regressing metastatic breast cancer in murine models.^[^ ^107^ ^]^

**Figure 10:**
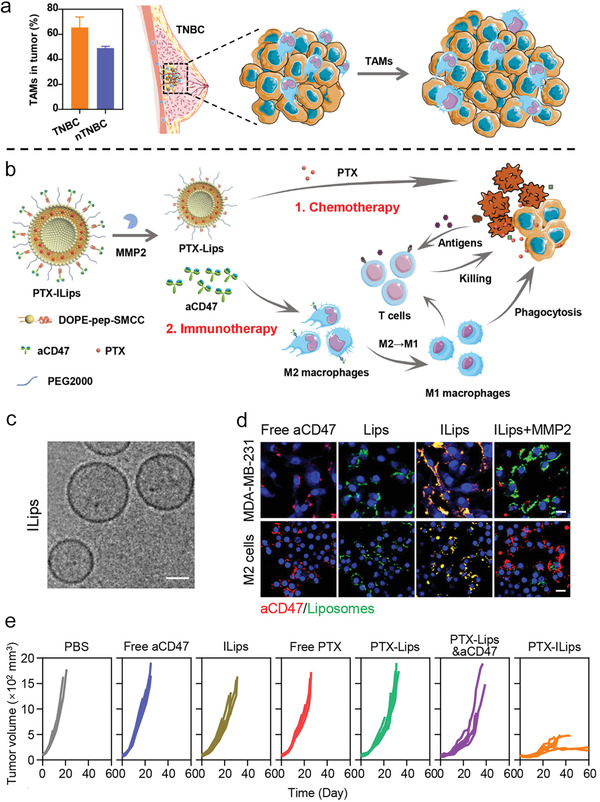
Checkpoint inhibitor anti‐CD47 mAbs and anti‐cancer small drugs co‐formed in the liposome nanoparticles enable targeting modulation of TAMs for significantly improved treatment of TNBC. Illustrated are a) increased TAMs in TNBC promoting tumor progression, b) the key components of engineered PTX‐ILips (immune liposomes) and therapeutic mechanisms against TNBC. c) A TEM image of ILips. Scale bar: 50 nm. d) Confocal images of cells treated with different formulations in the presence or absence of MMP2. Red indicates *α*CD47 and green indicates liposomes. Scale bar: 20 µm. e) Mice with established MDA‐MB‐231 tumor (around 100 mm^3^) received different treatments as indicated. Shown are the individual tumor growth curves. a–e) Reproduced with permission.^[^ ^112^ ^]^ Copyright 2021, American Chemical Society.

“Marker of self” CD47 ubiquitously expressing on all cells interacts with signal regulatory protein alpha (SIRP*α)* on macrophages and DCs,^[^ ^108^, ^109^ ^]^ avoiding the destruction by macrophage‐mediated phagocytosis. SIRP*α*‐CD47 inhibitory checkpoint plays a crucial role in protecting healthy cells, while tumor cells can significantly upregulate their CD47 expression compared to normal cells (two to six times) to escape from the recognition and destruction by macrophages. Therefore, block SIRP*α*‐CD47 inhibitory signal has become a promising strategy under clinical trials to treat patients with advanced malignancies and lymphoma.^[^ ^110^ ^]^ Administration of anti‐CD47 mAbs that block CD47‐SIRP*α* axis led to an increased frequency of M1‐like TAMs against tumor.^[^ ^111^ ^]^ In triple negative breast cancer (TNBC), increased TAMs were found promoting tumor growth (**Figure** **10**a), thus Zhang and colleagues engineered a liposome‐based nanoformulation (≈120 nm, Figure 10b,c) to target and modulate TAMs for enhanced treatment of breast cancer.^[^ ^112^ ^]^ Specifically, anti‐CD47 mAbs were conjugated on the surface of liposomes through a matrix metalloprotease 2 (MMP2) responsive phospholipid (Figure 10d), and the anti‐cancer small drug PTX was encapsulated into the phospholipid bilayers. Following intravenous administration, the nanoformulation exhibited increased accumulation at the CD47 expressing tumor site than liposomes without anti‐CD47mAbs. The responsive release of anti‐CD47 mAbs from the nanoformulation induced significantly enhanced polarization of M2 to M1 phenotype TAMs, with the aid of PTX‐induced ICD. The nanoformulation‐mediated‐TAM reprogramming in turn promoted effector T cell infiltration in tumor, thereby exhibiting superior anti‐tumor activity than free anti‐mAbs or PTX in a therapeutic MDA‐MB‐231 breast cancer model (Figure 10e).

**Figure 11:**
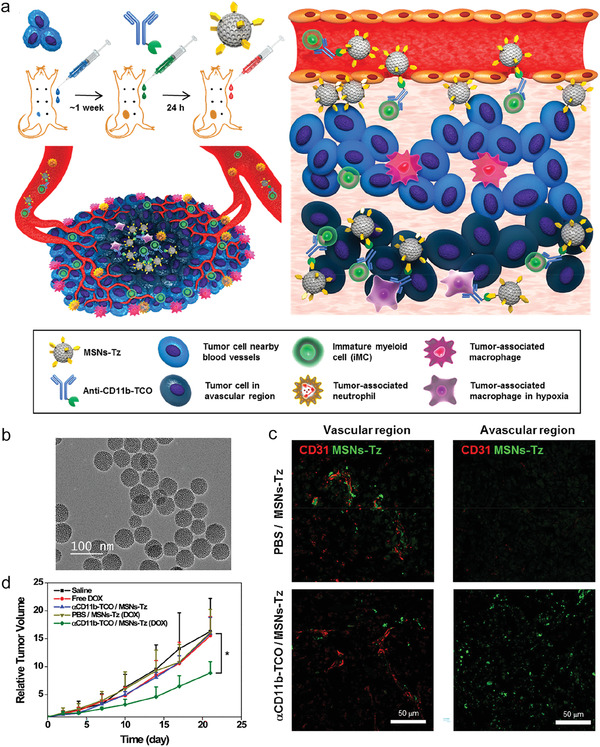
A biorthogonal system enables MSNs targeting CD11b+ monocytes circulating in blood that can be recruited to the deep inner site of tumor for improved cancer therapy. a) Illustrated is the specific targeting strategy of engineered MSNs to monocytes for deep tumor penetration. b) A TEM image of MSNs‐Tz. c) Confocal images of vascular and avascular regions of 4T1 tumor in mice received different treatments as indicated. Green: MSNs‐Tz; Red: CD31 expressed on endothelial cells of tumor blood vessels. d) Relative 4T1 tumor growth curves, normalized to the sizes when the mice received different therapeutic treatments as indicated. a–d) Reproduced with permission.^[^ ^114^ ^]^ Copyright 2019, American Chemical Society.

Monocytes circulating in blood are progenitors to TAMs and monocyte derived DCs. In response to the contexts of tissues and diseases, such as inflammatory environment in cancer, monocytes can be recruited to tissues and differentiate into terminal effector cells, such as M2‐like cells in tumor.^[^ ^113^ ^]^ Inspired by this fact, Hyeon and colleagues developed a new nanoformulation targeting circulating immune cells in blood for enhanced cancer therapy.^[^ ^114^ ^]^ As illustrated in **Figure** **11**a, anti‐CD11b mAbs conjugated with trans‐cyclooctene (Anti‐CD11b‐TCO) were intravenously injected in mice bearing 4T1 tumor, to target CD11b expressing monocytes circulating in blood. At 24 h post administration, mesoporous silica nanoparticles (MSNs) modified with tetrazines (MSNs‐Tz, around 50 nm, Figure 11b) were injected to tag CD11b+ cells through the biorthogonal click reaction between TCO and Tz. Compared to passive targeting approach, the monocyte‐mediated delivery allowed deeper penetration of MSNs in tumor (Figure 11c), resulting in enhanced therapeutic efficacy of MSNs‐carried chemodrug doxorubicin against 4T1 tumor (Figure 11d). It would be interesting to further explore whether the penetration depth of therapeutic nanoformulation affects the recruitment or differentiation of macrophages.

In a recently highlighted preprint^[^ ^115^, ^116^ ^]^ that has not been peer‐reviewed yet, Krummel and colleagues revealed the co‐dependency of TAMs and dysfunctional T cells in promoting tumor immune evasion. Dysfunctional T cells and TAMs engaged through persistent antigen specific synaptic interactions. TAM mediated suboptimal T cell receptor signaling leads to exhausted T cells. Synchronously, the factors produced by the dysfunctional T cells lead to the recruitment of extra monocytes, differentiating into TAMs. These findings suggest the need for new therapies that target both TAM and exhausted T cells to interrupt the positive feedback circuit, reversing immunosuppression and promoting anti‐tumor immunity. In addition, the authors used the cutting edge technique of ZipSeq (single cell spatial transcriptomics),^[^ ^117^ ^]^ to “label” the cells from the outer to inner area of tumor prior to scRNA sequencing. It was found that TAMs and CD8+ T cells sitting in the inner region of tumor present with more impaired function on antigen presentation and exhausted status, compared to those in outer region. Therefore, new targeting nanoformulations that can penetrate the hypoxic tumor core would improve the therapeutic outcomes of the immunodrugs targeting both TAMs and exhausted T cells. Tumor infiltrating immune cells are temporally, spatially, and functionally heterogeneous. With the advances in new technologies, such as CITE‐seq (cellular indexing of transcriptomes and epitopes by sequencing) and spatial transcriptomics, biologists and immunologists can uncover the mechanisms of tumor immunosuppression heterogeneity, offering new potential targets for cancer therapies. The combination of advanced nanoformulation technologies that enable specific site targeting would aid the development of new immunotherapeutics for better cancer treatment.

### 2.4 Nanoformulations Targeting NK Cells

NK cells that are innate cytotoxic lymphocytes can rapidly recognize and kill tumor cells, which are regulated by the joint signals from stimulatory and inhibitory receptors. In contrast to CTLs, prior antigen exposure is not required for NK cell natural cytotoxicity. NK cells express stimulatory receptors, such as NKG2D and NKp44L, being able to recognize the altered proteins expressed by cancer cells. Like CTLs, activated NK cells can directly eliminate cancer cells by the release of cytotoxic enzymes and cytokines. Tumor cells often have downregulated MHC class I molecules, to reduce the recognition and attack by antigen specific CTLs, while NK cells can exert their antigen unspecific cytolytic function on the cells with absent expression of MHC class I molecules that, in fact, suppress the cytotoxic activity of NK cells through the interaction with inhibitor KIRs (killer cell immunoglobulin‐like receptors), thereby minimizing the undesired destruction on healthy cells.^[^ ^118^ ^]^ NK cells are a unique subset among immune cells with powerful anti‐tumor efficacy, thus NK‐cell‐based cancer therapies are extensively explored under clinical trials.^[^ ^119^ ^]^

**Figure 12:**
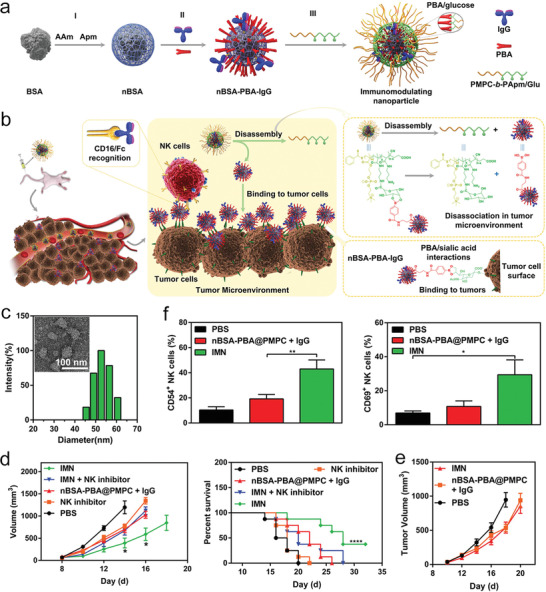
Multifunctional protein/polymer nanoformulation allows precise release of IgG antibodies at TME, targeting tumor surface antigen and enhancing ADCC‐mediated NK therapy. Illustrated are a) the fabrication procedure of immunomodulating nanoparticles (IMN) and b) the therapeutic mechanism of IMN in targeted cancer therapy. c) A TEM image (inset) and hydrodynamic size of IMN determined by dynamic light scattering (DLS). d) C57BL/6 mice bearing established B16F10 or e) SA‐depleted B16F10 tumor with a size of 50 mm^3^ received different treatments as indicated. Shown are average tumor growth curves and survival rate curves. f) CD54+ NK and CD69+ NK cell percentages of pre‐gated NK1.1+ cells in B16F10 tumor samples derived from mice received treatments as indicated. a–f) Reproduced with permission.^[^ ^121^ ^]^ Copyright 2019, Wiley‐VCH.

Antibody‐dependent cellular cytotoxicity (ADCC) is a crucial mechanism underlying NK cell therapy against antibody‐opsonized cancer cells, triggered by the interaction between the CD16 receptor expressed on NK cells and Fc fragment of antibodies. For example, following administration of anit‐GD2 mAbs that target the molecules highly expressed by neuroblastoma cells, allogenic NK cells were transferred into children with neuroblastoma, resulting in enhanced NK cells mediated ADCC therapeutic effect with 40% responders.^[^ ^120^ ^]^ To achieve spatial control of ADCC‐based therapeutics, Lu and colleagues engineered responsive nanoformulations for controlled release of encapsulated IgG antibodies in acidic tumor environment, thereby augmenting CD16‐Fc signaling mediated NK cell immunotherapy.^[^ ^121^ ^]^ As illustrated in **Figure** **12**a, BSA nanocapsules were first prepared by coating with a layer of crosslinked polymer. PBA（phenylboronic acid) and IgG were conjugated on the surface of BSA nanocapsules, followed by coating with PMPC‐b‐PApm/Glu. The resultant nanoformulation showed a small size of ≈50 nm (Figure 12c). The polymer layer of PMPC‐b‐PApm/Glu displayed responsive release of inner core particles in the presence of elevated proton or sialic acid (SA). These properties enabled nBSA‐PBA‐IgG core particles precisely released at acidic TME following passive accumulation, and then bound with SA overexpressed on the surface of tumor cells (Figure 12b). Tumor infiltrating NK cells (Figure 12f) were activated by interacting with Fc fragment of IgG tagged on tumor cells, resulting in enhanced ADCC‐mediated regression of 4T1 and B16F10 tumor (Figure 12d,e).

**Figure 13:**
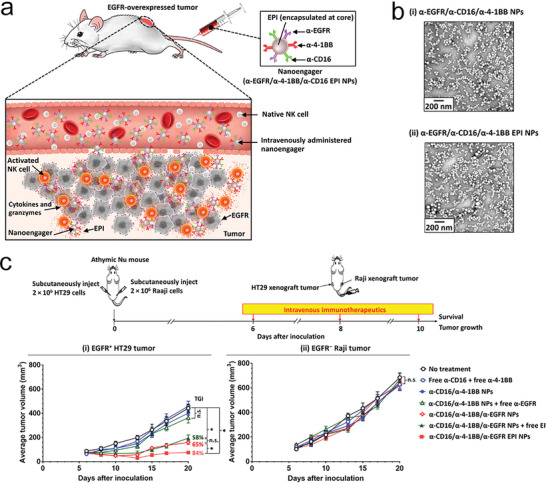
Trispecific NK cell nanoengagers potentiate NK cell‐medicated anti‐tumor activity. a) Illustrated is the therapeutic mechanism of nanoengager against EFGP overexpressed tumor post intravenously administrated into mice. b) TEM images of different nanoparticles as indicated. c) Nu mice were subcutaneously injected with EGFR+ HT29 cells on one side and Raji cells on the other side on day 0, and then received different immunotherapeutic treatments (as indicated) on days 6, 8, and 10. Shown are the average tumor growth curves. a–c) Reproduced with permission.^[^ ^123^ ^]^ Copyright 2020, American Association for the Advancement of Science.

To potentiate NK cell mediated anti‐tumor activity, therapeutic antibodies have been recently engineered and produced with single‐chain multifunctional fragments that target NK cell receptors together with tumor antigen, termed bispecific or trispecific killer cell engagers (abbreviated as BiKEs or TriKEs).^[^ ^122^ ^]^ Instead of engineering antibodies, Wang and colleagues designed biocompatible PEG‐PLGA nanoparticles functionalized with trispecific killer cell engagers, consisting of mAbs targeting activation of NK‐cell receptor CD16 and 4‐1BB, and human tumor antigen EGFR (around 100 nm in diameter, **Figure** **13**a,b).^[^ ^123^ ^]^ Additionally, a therapeutic anti‐cancer drug of epirubicin (EPI) can be encapsulated into the nanoengagers for combined therapy (Figure 13a,b). Mechanically, the nano‐TriKEs allowed spatiotemporal regulation on the engagement of NK cells and tumor cells by specifically targeting EGFR‐overexpressing tumor cells and potentiating NK‐cell activation via CD16 and 4‐1BB receptors. The activated NK cells secreted cytotoxic cytokines and enzymes to kill the engaged cancer cells (Figure 13a). The therapeutic effect of the nanoengagers was evaluated in multiple tumor models, including EGFR expressing mouse breast cancer A431, human breast cancer MB468 xenograft models, and human colorectal adenocarcinoma EGFP+ HT‐29 and lymphoma model EGFP‐Raji dual xenograft models in T cell‐deficient mice (Figure 13c). The results revealed tumor specificity and superior killing potency to free antibodies (Figure 13c). The combination of nano‐TriKEs and EPI demonstrated significantly improved cancer therapy. It would be interesting to compare the anti‐tumor efficacy and safety of the nano‐TriKEs with therapeutic TriKEs antibodies. Furthermore, the therapeutic effect of this platform in humanized mouse model would provide additional preclinical evidence for its clinical translation in future.

There are several studies of nanoformulations targeting other types of immune cells ex vivo, such as neutrophils,^[^ ^10^, ^124^, ^125^, ^126^ ^]^ before infusion back to animals for potent cancer immunotherapy, which is out of our scope on in vivo targeting nanoformulations. Thus, the details of these studies are not introduced here. The summary of exemplified nanoformulations for targeted cancer immunotherapy is listed in **Table** **1**. Inorganic nanoformulations, such as silica nanoparticles, can be precisely engineered with various sizes, porosity, shape, and topology. Most inorganic candidates are relatively stable under physiological conditions, while potential toxicity caused by mental ions formulated in organic nanoparticles is the key limiting factor for their clinical development.^[^ ^127^ ^]^ In contrast, organic nanoformulations, particularly lipid and polymeric nanoparticles, offer advantages in fabrication simplicity, biocompatibility, and easy modulation of biological properties.^[^ ^128^ ^]^ For these reasons, the advanced and promising candidates under clinical trials in cancer patients are often organic nanoformulations, which are described in next section.

**Table 1:** The most recent advanced nanoformulations targeting immune cells for cancer therapy

Nanomaterials	Size	Targeting subsets of immune cells	Targeting strategies	Immuno‐cargo molecules	Tumor model	Mechanistic insights	Ref.
Lipid/peptide nanodiscs	10 nm	dLN‐DCs	Small size	Antigen (SIINFEKL; Adpgk; 27+M30+TRP2) +CpG	MC38, B16F10	Cytosolic pathway mediated cross presentation for enhanced CTLs against tumor	[37]
PC7A polymer nanoparticles	30 nm	LN‐resident CD8*α*+ DC	Small size	Antigen OVA; HPV E7 peptide	B16‐OVA, TC‐1	STING pathway activation and cross presentation for induction of potent CTLs against tumor.	[40]
Azole/PEI	50–100 nm	dLN‐DCs	Small size	OVA; tumor cell lysis	B16‐OVA; MC38	Same as above	[43]
Peptide/TLR7/8a micelles	20 nm	dLN‐cDC1s (S.C.); monocyte‐derived DCs (I.V.)	Small size	Antigen (7 MC38 neoantigen; HPV E6/E7; self‐antigen Trp1)+TLR7/8a adjuvant	MC38, TC‐1, B16F10	I.V. administration targeted monocyte‐derived DCs for induction of stem‐like CD8+ T cells for potent anti‐tumor activity.	[49, 50]
CpG/shRNA nanoparticles	250 nm	dLN‐DCs	Small size	Antigen (SIINFEKL;Adpgk)+Adjuvant (CpG+shRNA)	MC38	Dual adjuvants delivered antigen and activated dLN‐DCs concurrently, triggering potent anti‐tumor T cell immunity.	[51]
Ionizable lipid nanoparticles	100 nm	dLN‐DCs	Small size	mRNA encoding tumor antigens (OVA and E7)	B16‐OVA, TC‐1	Heterocyclic lipids promoted mRNA transfection and STING activation for enhanced anti‐tumor immunity.	[53]
Nanoemulsion	200 nm	Splenic Clec9a+cDC1s	Anti‐Clec9a mAb	HPV E76/E7 protein, B16F10 neoepitopes	TC‐1, B16F10	Active targeting delivery of tumor antigens to cross presenting DCs for high magnitude of T cell immunity against tumor.	[54]
Polymer/Mn^2+^	150 nm	LN‐DCs	Small size	OVA+Nod1 agonist	B16‐OVA	Nanoformulation modulated DC activating by cross presentation of antigens and Nod1 receptor signaling against tumor.	[62]
Cationic cholesterol lipid nanoparticles	70 nm	LN‐DCs	Small size	OVA+CpG		Nanoformulation activated DC via TLR signaling promoted cross presentation capability for enhanced T cell priming and anti‐tumor activity.	[63]
Mannan/PLA/PEI polymer nanoparticles	100 nm	LN‐DCs	Small size	Antigen (OVA, MC38 tumor lysis+adjuvants (TLR4 agonist of mannan+CpG)	B16‐OVA;MC38	Dual adjuvants activated dLN‐DCs concurrently, triggering potent anti‐tumor T cell immunity.	[64]
Albumin‐stabilized nanoemulsion	330 nm	LN‐DCs	Deformability	Antigen (SIINFEKL)	E.G7/OVA	The deformable nanoemulsion enabled drainage to LNs for potent T cell priming against tumor.	[30]
Liposome	160 nm	Tumor‐DCs	EPR effect	R837 (TLR7 agonist) +sonosensitizer (HMME)	4T1, CT26	R837 together with SDT‐induced ICD promoted DC activation and tumor antigen presentation for enhanced therapeutic effect.	[68]
Cell membrane/PLGA	160 nm	Tumor‐Clec9a+cDC1s	CBP‐12 peptide targeting Clec9a	2′3′‐cGAMP (STING agonist)	TC‐1, B16F10	STING pathway‐mediated cDC1 activation in tumor for antigen cross presentation, priming polyfunctional CD8+ T cells against tumor.	[74]
PLGA/PEG nanoparticles	270 nm	(PD‐1+) CD8+ T cells	Anti‐CD8a, anti‐PD‐1 mAbs	TGF*β*R1 inhibitor (SD‐208), TLR7/8 agonist (R848)	MC38, B16F10	Sustained release of SD‐208 efficiently obstructed TGF*β* signaling mediated immunosuppression, restoring the function of effector CD8+ T cells against tumor; Delivered R848 restored innate immunity.	[84]
Protein self‐linked nanogels	80–130 nm	Primed T cells	Anti‐CD45 mAbs and reducing agents	IL‐15 super agonist (IL‐15Sa)	B16F10, U‐87	IL‐15Sa precise release to primed T cells substantially augmented T cell expansion against tumor.	[85]
Fucoidan/dextran/iron oxide nanoparticles	145 nm	PD1+ CD8 T cells	External magnetic field	Anti‐CD3, anti‐CD28, anti‐PD‐L1 mAbs, fucoidan	4T1, CT‐26	Precise co‐delivery of anti‐PD‐L1/T cell activator mAbs enhanced tumor infiltrating T cells and reduced Tregs, for improved anti‐tumor effect.	[86]
Phospholipid nanoparticles	50 nm	Tumor CD8+ T cells	EPR effect	OX4O mRNA	B16F10, A20	Increased expression of OX‐40 receptor on T cells enhanced anti‐OX‐40 mAbs‐mediated T cell activation and proliferation to combat tumors.	[88]
PbAE polymer nanoparticles	155 nm	CD3+ T cells	Anti‐CD3 mAbs+nuclear transport peptide	Plasmid DNA antigen	Eµ‐ALL01	Increased expression of leukemia‐specific 194‐1BBz CAR enhanced T cell‐mediated regression of leukemia.	[89]
Exosome nanoparticles	100 nm	CD3+ T cells	Anti‐CD3 mAbs	Anti‐CD3, anti‐EGFR mAbs	MDA‐MB‐468	Linking CD3+ adoptive T cells and EGFR+ tumor cells promoted T cell anti‐tumor immunity.	[93]
Lipid nanoparticles	Not provided	CD3+ T cells	Anti‐CD3 mAbs	Anti‐CD3, BCMA, CS1,CD38 mAbs	BCWM.1; MM.1S	Multiple nanoengagers increased half‐time of mAbs and tumor accumulation and prevented tumor escape.	[94]
PEG/Gold nanorods	13 × 47 nm	CAR T cells, TCR engineered T cells in tumor	ERP effect	‐	K562, MDA‐MB‐468, B16F10	Thermal control production of IL15Sa or NKG2D stimulatory receptors by engineered adoptive *α*CD19 CAR T or Pmel‐1 specific T cells against tumors.	[95]
*β*‐cyclodextrin nanoparticles	30 nm	TAMs	EPR effect	R848	MC38, B16F10	Altering suppressive M2‐like phenotype to an anti‐tumor M1 phenotype promoted anti‐tumor activity.	[105]
Albumin	143 nm	TAMs	EPR effect	PI3K*γ* inhibitor (IPI‐549)	4T1 and MMTV‐PyMT spontaneous breast cancer	Remodulating macrophages in both tumor sites and lymph nodes, polarizing M2 to M1, and increasing T cell immunity for long term tumor remission	[107]
PbAE polymer	100 nm	TAMs	EPR effect	IRF5/IKK*β*mRNA	B16F10, ID8, glioma	Genetically reprogramming M2 with increased IRF5/IKK*β* expression regulated M1 politization for enhanced T cell tumor infiltration.	[31]
Liposome	120 nm	TAMs	Anti‐CD47 mAbs	Anti‐CD47 mAbs+PTX	MDA‐MB‐231	Responsive release of anti‐CD47 mAbs promoted polarization of M2 to M1 phenotype for enhanced cancer treatment in combination with PTX.	[112]
Tz‐silica nanoparticles	50 nm	CD11b+ monocytes	Click chemistry interaction	Doxorubicin	4T1	Monocyte‐mediated delivery allowed deep tumor penetration for enhanced therapeutic efficacy of drugs.	[114]
Protein/polymer nanoparticles	50 nm	NK cells	IgG	IgG	B16F10, 4T1	Fc‐CD16 interaction enhanced NK cell‐mediated tumor regression.	[121]
PEG‐PLGA	100 nm	NK cells	Anti‐ EGFR antibody, anti‐CD16, anti‐4‐1BB mAbs	Anti‐ EGFR antibody, anti‐CD16, anti‐4‐1BB mAbs	B16F10, A431, MB468, HT29	This multifunctional nanoparticle potentially activated NK cells against tumor	[123]

## 3 Clinical Development of Nanoformulations in Targeted Cancer Immunotherapy

The immuno‐drugs in a manner of nanoformulations have demonstrated encouraging preclinical therapeutic outcomes that are not achieved by free immuno‐molecules in solution, supporting their success moving from the bench to clinical trials (**Table** **2**). For example, poly‐ICLC, an agonist for TLR3 containing double‐stranded RNA (dsRNA) protected by polylysine, is a promising candidate at advanced clinical development. In 2011, Sékaly and colleagues reported a small‐scale human study on the innate immune response to poly‐ICLC.^[^ ^129^ ^]^ Following subcutaneous administration, poly‐ICLC showed upregulated genes associated with innate cells pathways, particularly type I IFN and inflammasome signaling, similar to those induced by microbials. Given the potent adjuvancity and DC targeting capability, poly‐ICLC alone, conjugation with peptide/protein antigens, or combination with immune checkpoint inhibitors (ICIs), has been studied in phase 1/2 clinical trials to assess its safety and efficacy in patients with colon cancer, triple‐negative breast cancer, ovarian, or glioblastoma (Table 2).The preliminarily promising results demonstrated tolerability and anti‐tumor activity.^[^ ^130^ ^]^ ISCOMs (immune‐stimulating complexes) that consist of saponin QuliA adjuvant, phospholipid, and cholesterol are cage‐like nanoformulation adjuvants with a diameter of 40–60 nm,^[^ ^131^ ^]^ which can carry peptide/protein antigens and target APCs in lymphatic system. ISCOMs were first described in 1984,^[^ ^132^ ^]^ representing another type of well‐known adjuvants under clinical trials.^[^ ^133^ ^]^ For example, the combination of ISCOMs and tumor antigen New York esophageal squamous cell carcinoma‐1 (NY‐ESO‐1) protein,^[^ ^134^ ^]^ was found safe and highly immunological with high‐titer antigen specific antibody responses and a broad range of T cell immunity in participants with resected NY‐ESO‐1 positive melanoma in a phase I clinical trial.^[^ ^135^ ^]^ The patients intramuscularly vaccinated with ISCOMs/NY‐ESO‐1 appeared improved relapse‐free survival compared to those received placebo or NY‐ESO‐1 antigen alone.^[^ ^136^ ^]^ In a phase 2 clinical trial, the vaccine did not impact survival endpoints of patients with fully resected melanoma,^[^ ^137^ ^]^ of which the downregulation of antigen and/or HLA class I molecules might lead to the immune escape, though vaccination induced strong antigen specific immunity. Further development of ISCOMs+NY‐ESO‐1 cancer vaccine was terminated.

**Table 2:** Summary of clinical studies of nanoformulations targeting immune cells for cancer therapy

Nanomaterials	Targeting subsets of immune cells	Administration approach	Immuno‐cargo molecules	Models	Product name	Status	Stage (Clinicaltrials.gov identifier)
Polylysine/carboxymethylcellulose	DCs	i.m.	Polyinosinic‐polycytidylic acid (Poly I:C)	Multiple cancers	Poly ICLC	Completed/recruiting/terminated/completed/completed/completed	Phase‐1/2 (NCT00374049/ NCT02834052/ NCT03721679/ NCT00986609/ NCT02166905/ NCT02078648)
Liposome	APCs	i.m.	Adjuvant (saponin Quil A) and antigen (NY‐ESO‐1)	Melanoma	NY‐ESO‐1 ISCOMATRIX	Completed/competed	Phase‐2 (NCT00199901/ NCT00518206)
Liposome	APCs	i.m.	Adjuvants (MPL + QS‐21 + CpG) and tumor antigens	Multiple cancers	AS15	Completed/completed/completed/terminated/completed/completed/completed/unknown/terminate/terminated/completed/completed/terminated/completed/terminated	Phase‐1/2/3 (NCT00952692/ NCT01498172/ NCT02364492/ NCT01435356/ NCT00148928/ NCT01853878/ NCT00058526/ NCT00086866/ NCT00796445 NCT00480025/ NCT01266603/ NCT00140738/ NCT01159964/ NCT01149343/ NCT01220128)
Liposome	dLN‐APCs	s.c.	TAA andMPL	Multiple cancers	L‐BLP25	Terminated/completed/completed/completed/withdrawn/completed/completed/completed/terminated/completed/completed/completed/terminated/terminated	Phase‐1/2/3 (NCT01423760/ NCT01462513/ NCT01094548/ NCT01496131/ NCT01731587/ NCT01507103/ NCT00157196/ NCT00960115/ NCT01015443/ NCT00157209/ NCT00828009/ NCT00409188/ NCT00925548/ NCT02049151)
Cholesteryl pullulan nanogels	APCs	s.c.	HER‐2/NY‐ESO‐1	Multiple cancers	CHP‐HER2; CHP‐NY‐ESO‐1	Completed	Phase‐1 (NCT00291473)
Liposome (DPX)	APCs	s.c.	Antigens (TAA peptides) and adjuvants (polynucleotide and Montanide ISA51 VG)	Multiple cancers	DPX‐0907	Completed	Phase‐1 (NCT01095848)
Liposome (DPX)	APCs	s.c.	As above	Multiple cancers	DPX‐Survivac	Recruiting/completed/active, not recruiting/recruiting/completed/active, not recruiting/active, not recruiting/recruiting/recruiting	Phase‐1/2 (NCT04895761/ NCT01416038/ NCT02785250/ NCT03029403/ NCT03332576/ NCT03836352/ NCT03349450/ NCT05243524/ NCT04920617)
Liposome (DPX)	APCs	s.c.	E7	HPV related head and neck, cervical or anal cancer	DPX‐E7 vaccine	Active, not recruiting	Phase‐1/2 (NCT02865135)
VLP	APCs	s.c.	Antigen (Melan‐A peptides) and adjuvant (CpG‐A)	Melanoma	MelQbG10	Completed/completed/completed/completed/completed	Phase‐1/2 (NCT00306566/ NCT00306514/ NCT00306553/ NCT00651703/ NCT00324623)
Liposome	APCs	s.c.	Antigen (MUC1) and adjuvant (PET lipid A)	Solid tumors	ONT‐10	Completed/completed/completed	Phase‐1 (NCT01556789/ NCT01978964/ NCT02270372)
DMS‐5000/liposome	DCs	i.v.	Antigens (Gp100, melan A/MART‐1, tyrosinase) and adjuvants (IFN‐*γ*)	Melanoma	Lipovaxin‐MM	Completed	Phase‐1 (NCT01052142)
Liposome	APCs	s.c.	Tumor‐derived antigen and IL‐2	Lymphoma	‐	Completed	Phase‐1 (NCT00020462)
Liposome	Peripheral immune cells	i.v.	Plasmid DNA complex	Leukemia	JVRS‐100	Completed	Phase‐1 (NCT00860522)
Liposome	APCs	s.c.	Extract of a person's cancer cells and IL‐2	Leukemia	Oncoquest‐CLL	Active, not recruiting	Phase‐1 (NCT01976520)
Liposome	T cells	s.c.	IL‐2	Melanoma	‐	Completed	Phase‐2 (NCT00004104)
Heat‐shock protein	APCs	s.c.	Autologous tumor antigens	Multiple tumors	gp96	Unknown/completed/unknown	Phase‐1/2 (NCT02317471/ NCT02122822/ NCT02133079)
Lipid	APCs	s.c.	Antigens (peptides) and adjuvants (CpG‐7909)	Multiple solid tumors	ELI‐002 2P	Recruiting	Phase‐1 (NCT04853017)
PLGA	iNKT and APCs	i.v.	NY‐ESO‐1 and iNKT activator threitolceramide‐6	Advanced solid tumor	PRECIOUS‐01	Recruiting	Phase‐1 (NCT04751786)
PEI	dLN‐APCs	i.m.	DNA encoding neuroblastoma‐associated antigen fused with potato virus X coat protein	Neuroblastoma	DNA‐PEI polyplex	Recruiting	Early Phase‐1 (NCT04049864)
IL‐15 superagonist complex nanogel	CD8+ T cells and NK cells	i.v./i.p./s.c.	IL‐15N72D + rmIL‐15R*α*‐Fc	Hematological malignancies/ Solid tumors	ALT‐803	Completed/active, not recruiting/withdrawn (trial not initiated)/unknown/active, not recruiting/active, not recruiting/active, not recruiting/completed/terminated/completed/completed/unknown/active, not recruiting/withdrawn/completed/recruiting/recruiting	Phase‐1/2/3 (NCT01727076/ NCT03563157/ NCT03647423/ NCT03586869/ NCT03387098/ NCT03329248/ NCT02890758/ NCT02523469/ NCT02559674/ NCT02384954/ NCT01885897/ NCT03127098/ NCT02099539/ NCT03054909/ NCT03365661/ NCT01946789/ NCT03050216/ NCT02138734/ NCT03022825)
Lipoplex	Splenic cDCs, pDCs, and macrophages	i.v.	mRNA encoding TSAs	Melanoma Stage III/IV; unresectable melanoma	BNT111	Recruiting/active, not recruiting	Phase‐1/2 (NCT04526899/ NCT02410733)
Lipoplex	As above	i.v.	As above	Metastatic castration‐resistant prostate cancer	BNT112	Recruiting	Phase‐1/2 (NCT04382898)
Lipoplex	As above	i.v.	mRNA encoding HPV16 E6/E7	HPV16+ head and neck squamous cell carcinoma	BNT113	Recruiting	Phase‐2 (NCT04534205)
Lipoplex	As above	i.v.	mRNA encoding TAAs	Triple‐negative breast cancer	BNT114	Active, not recruiting	Phase‐1 (NCT02316457)
Lipoplex	As above	i.v.	As above	Ovarian cancer	BNT115	Active, not recruiting	Phase‐1 (NCT04163094)
Lipoplex	As above	i.v.	As above	Non‐small‐cell lung cancer	BNT116	Not yet recruiting	Phase‐1 (NCT05142189)
DOTAP Liposome	As above	i.v.	Total tumor mRNA and pp65 full length LAMP mRNA	Pediatric high‐grade glioma/glioblastoma	RNA‐LP	Recruiting	Phase‐1 (NCT04573140)
Lipoplex	As above	i.v.	mRNA encoding personalized cancer vaccine	Multiple resected/advanced cancers	BNT122	Recruiting/active, not recruiting/active, not recruiting/withdrawn/active, not recruiting	Phase‐1/2 (NCT04486378/ NCT04161755/ NCT03815058/ NCT04267237/ NCT03289962)
Lipoplex	CAR‐T cells	i.v.	mRNA encoding CLDN6	Solid tumor	BNT211	Recruiting	Phase‐1/2 (NCT04503278)
LNP	NK cells	i.v.	mRNA encoding anti‐Claudin 18.2 antibodies	Solid tumor	BNT141	Recruiting	Phase‐1/2 (NCT04683939)
Cationic polymer/lipid formulation	Tumor‐T cells, tumor cells	i.v.	mRNA encoding CD3xCLDN6 bispecific antibodies	Solid tumor	BNT142	Recruiting	Phase‐1/2 (NCT05262530)
LNP	T cells	i.v.	mRNA encoding optimized IL‐2	Solid tumor	BNT151	Recruiting	Phase‐1/2 (NCT04455620)
LNP	As above	i.v.	mRNA encoding IL‐2 and mRNA encoding IL‐7	Solid tumor	BNT152 and BNT153	Recruiting	Phase‐1 (NCT04710043)
LNP	dLN‐APCs	i.m.	mRNA encoding personalized cancer vaccine	Multiple cancers	mRNA‐4650	Terminated	Phase‐1/2 (NCT03480152)
LNP	As above	i.m.	As above	Solid tumor/melanoma	mRNA‐4157	Recruiting/active, not recruiting	Phase‐1/2 (NCT03313778/ NCT03897881)
Lipopolyplex	As above	s.c.	As above	Multiple advanced digestive system cancers	‐	Unknown	Not Applicable (NCT03468244)
LNP	As above	i.m.	mRNA encoding KRAS mutated proteins	Multiple KRAS mutant advanced/metastatic cancers	mRNA‐5671	Active, not recruiting	Phase‐1 (NCT03948763)

AS15 is a liposomal nanoformulation adjuvant comprised of monophosphoryl lipid A (MPL), QS‐21, and CpG 7909. A phase 2 clinical study (NCT00086866) revealed that AS15 combined with melanoma antigen family A, 3 (MAGE‐A3) tumor antigen induced strong clinical activity (robust MAGE‐A3 specific antibody and T cell immune response) in patients with melanoma,^[^ ^138^ ^]^ thus phase 3 studies of MAGE‐A3+AS15 therapeutic vaccines were initiated in patients with surgically resected melanoma (NCT00796445) and non‐small‐cell lung cancer (NSCLC) (NCT00480025). The clinical outcomes revealed that MAGE‐A3+AS15 vaccine alone did not show therapeutic efficacy in these cancer patients and the development of the therapeutic vaccine for use in these malignancies has been stopped.^[^ ^139^, ^140^ ^]^ A phase 2 study of MAGE‐A3+AS15 vaccine in patients who experienced surgical removal of bladder cancer was also terminated (NCT01435356). In parallel, the combination therapy of MAGE‐A3+AS15 vaccine and IL‐2/ICIs was evaluated in clinical development. A phase 2 clinical study (NCT01266603) proved the safety profile of the combination therapy similar to that of high dose IL‐2 monotherapy and showed that vaccination provided strong anti‐tumor activity in melanoma patients.^[^ ^141^ ^]^ The clinical immune monitoring data suggested a potential treatment strategy by combining MAGE‐A3+AS15 vaccine and ICIs. Another completed phase 1 study investigated the Bacillus Calmette‐Guerin (BCG) modulation of MAGE‐A3+AS15 vaccine in the patients with bladder cancer (NCT01498172, Table 2).

Beyond MAGE‐A3 tumor antigen, AS15 has been under clinical development in combination with other types of cancer antigens for the treatment of patients with other malignancies, such as combination with HER2) protein antigen for treating HER2+ metastatic breast cancer (phase 1, NCT00058526;^[^ ^142^ ^]^ phase 1/2, NCT00140738;^[^ ^143^ ^]^ phase 1, NCT00952692^[^ ^144^ ^]^), preferentially expressed antigen in melanoma (PRAME) protein for treating NSCLC (phase 1, NCT01159964;^[^ ^145^ ^]^ phase 2, NCT01853878) and melanoma (phase 1/2, NCT01149343^[^ ^146^ ^]^), Wilms' tumor 1 (WT1) antigen for treating WT1+ breast cancer (phase 2, NCT01220128, terminated), P501 antigen for treating prostate cancer (phase 1, NCT00148928), and MAG‐Tn3 for HER2‐ breast cancer (phase 1, NCT02364492^[^ ^147^ ^]^). Despite the observed acceptable safety profile and immunogenicity, GSK decided to terminate the clinical development of cancer vaccines consisting of recombinant proteins and AS15, considering the negative results of the AS15 adjuvant platform in two phase 3 clinical trials of MAGE‐A3 therapeutics.

Liposomal nanoformulation L‐BLP25, cancer associated mucin 1 (MUC1) vaccine, is another well investigated therapeutic candidate in phase 1/2/3 clinical trials (Table 2), of which the clinical results demonstrated that L‐BLP25 was well tolerated and capable of inducing T cell immune response in patients with NSCLC.^[^ ^148^ ^]^ In a phase 2b clinical study, patients vaccinated subcutaneously with L‐BLP25 appeared a potential benefit in survival time compared to those only received best supportive care.^[^ ^149^ ^]^ Given these promising clinical outcomes, a phase 3 trial was initiated to investigate whether L‐BLP25 could improve the survival time of patients after chemoradiotherapy, while the preliminary clinical results did not show significant difference between patients received vaccination and placebo.^[^ ^150^ ^]^ The clinical development of this product has been stopped.

A nanogel formed by cholesteryl pullulan (named CHP, 25 nm in diameter^[^ ^151^ ^]^) was well examined in protein antigen delivery, and the resulting CHP‐antigen vaccine formulations were capable of targeting APCs and inducing antigen specific T cell immunity against murine tumor.^[^ ^152^ ^]^ A protein cancer vaccine of CHP‐HER2 was assessed in a clinical study in patients with HER2+ cancers, showing clinical response with HER2‐specific T cell immunity.^[^ ^153^ ^]^ Vaccination of patients with CHP‐NY‐ESO‐1 vaccine also induced antigen specific T cell response^[^ ^154^ ^]^ (Table 2).

Instead of carrying recombinant protein antigens, DPX‐0907 is a strongly immunogenic liposomal vaccine formulation (120 nm in diameter) delivering multiple HLA restricted peptides and polynucleotide‐based adjuvants. DPX‐0907 was capable of inducing multifunctional effector T cell immune response against antigens in patients with breast, ovarian, or prostate cancer (phase 1, NCT01095848^[^ ^155^ ^]^). The vaccine formulation of DPX‐ Survivac using surviving HLA peptides also showed induction of robust polyfunctional T cell immunity in ovarian cancer patients (phase 1, NCT01416038^[^ ^156^ ^]^). These promising clinical activities have led to the further clinical evaluation of this peptide cancer vaccine platform as monotherapy or combination therapy with cyclophosphamide/radiation/IDO1 inhibitor in patients with HER2‐ breast cancer (phase 1, NCT04895761), ovarian, peritoneal carcinoma, or fallopian tube cancer (phase 1/2, NCT02785250; NCT03029403; phase 1, NCT03332576; phase 2, NCT05243524), hepatocellular carcinoma, NSCLC, bladder cancer (phase 2, NCT03836352), large B cell lymphoma (phase 2, NCT03349450 and NCT04920617). DPX‐E7 vaccine currently is under phase1/2 clinical trial (NCT02865135) to evaluate its safety profile and therapeutic efficacy in patients with HPV16 associated malignancies (head and neck, cervical or anal cancer).

MelQbG10 is another type of peptide‐based vaccine,^[^ ^157^ ^]^ consisting of 1) highly immunogenic virus‐like particles (VLP, 30 nm in diameter) formed by shell proteins derived from bacteriophage Qbeta, and immunostimulatory G10, unmodified A‐type CpG, and 2) peptides derived from antigen Melan‐A/MART‐1(melanoma antigen recognized by T cells 1) that is expressed in advanced melanoma.^[^ ^158^ ^]^ The first phase 1/2 clinical trial of MelQbG10 revealed that immunization with MelQbG10 was well tolerated and capable of inducing CD8+ T cell immunity in patients with melanoma.^[^ ^159^ ^]^ In combination of additional adjuvants, MelQbG10 vaccine increased memory and effector phenotypic T cell response in patients with melanoma (phase 2, NCT00651703^[^ ^157^ ^]^). The combination therapy of MelQbG10 and chemotherapy (cyclophosphamide, fludarabine phosphate) was evaluated in melanoma patients recruited under a phase 1 study (NCT00324623).

Several other types of liposome, heat shock protein, lipid, PLGA or PEI‐based vaccine, or immune‐stimulator nanoformulations formed by different components were evaluated in phase1/2 clinical trials (such as ONT‐10, Lipovaxin‐MM,^[^ ^160^ ^]^ JVRS‐100, ELI‐002 2P and PRECIOUS‐01^[^ ^161^ ^]^), which are summarized in Table 2.

The recombinant IL‐15 superagonist complex nanogel that targets NK and T cells as introduced in the section above, named ALT‐803, has been evaluated under phase 1/2/3 clinical trials (as listed in Table 2) to assess the safety and efficacy for treating patients with advanced solid tumors (such as NSCLC, melanoma, renal carcinoma, colon cancer, and breast cancer), or leukemia. Phase I clinical studies revealed that ALT‐803 was well tolerated, increased expansion of NK cells (NCT01727076^[^ ^162^ ^]^) and CD8+ T cells (NCT01885897^[^ ^163^ ^]^), and promoted anti‐tumor activity (NCT02523469,^[^ ^164^ ^]^ NCT02138734^[^ ^165^ ^]^) in cancer patients. Recent clinical results from a phase 2/3 trial (NCT03022825) of ALT‐803 combined with BCG showed exceeded clinical response, safety, and efficacy, compared to available options for patients with bladder cancer.^[^ ^166^ ^]^ These encouraging clinical outcomes support further development of ALT‐803 product with numerous clinical trials underway, particularly in the combination with chemotherapy, NK cell therapy, cancer vaccines, radiation therapy, and/or ICIs.

The preferred nanoformulations that are currently active under clinical studies are LNP‐mRNA technology, of which lipoplex enables targeted delivery of therapeutic mRNA cancer vaccines to APCs in lymph organs, and subsequent proliferation of abroad T cell immunity in preclinical^[^ ^167^ ^]^ and clinical^[^ ^168^ ^]^ settings. Sahin and colleagues revealed that the surface charge of lipoplexes determined their biodistribution post intravenous administration in mice.^[^ ^167^ ^]^ Near neutral lipoplexes were found precisely and effectively targeting splenic CD11c+ cDCs, plasmacytoid DCs (DCs), and macrophages, which promoted encoded antigen expression in APCs and triggered highly potent innate and adaptive immunity against aggressive tumors in mice. This mRNA vaccine approach has been extensively evaluated under phase 1 clinical trials in cancer patients, such as the products BNT111, BNT112, and BNT113 encoding TSAs, BNT114, BNT115, and BN116 encoding TAAs, and BNT122 encoding personalized cancer vaccines (Table 2). The clinical study results of BNT111 (NCT02410733) recapitulated the observations in mice. The translation of mRNA encoding melanoma antigen was found in DCs resident at lymphoid organs, inducing strong polyclonal functional CD4+ and CD8+ T cells against antigens in patients with advanced melanoma.^[^ ^168^ ^]^ BNT111 monotherapy, or combination therapy with anti‐PD‐1 mAbs demonstrated tumor regression in patients. Another lipoplex‐mRNA product named BNT211 encodes antigen claudin‐6 (CLDN6) that is a tetraspanin membrane protein integral to tight junction formulation and widely expressed in tumors, which empowered proliferation of adoptive CLDN6‐CAR‐T cells to regress large CLDN6+ solid tumors in mouse models.^[^ ^169^ ^]^ The preliminary clinical data showed encouraging safety profiles and expansion of CLDN6‐CAR‐T cells in patients with CLDN6+ tumors.^[^ ^170^ ^]^ Along with these therapeutic mRNA cancer vaccines, BioNTech's product pipelines on clinical program include LNP‐mRNA products encoding antigen specific therapeutic mAbs (BNT141 and BNT142) or cytokines (BNT151, BNT152, and BNT153), which are currently being assessed in patients with solid tumors. In parallel, Moderna is investigating the applicability of mRNA immune medicines for the treatment of cancer patients, such as mRNA‐4650 and mRNA‐4157 therapeutic mRNA vaccines. The unique amine‐lipid structure endowed Moderna's nanoformulation with enhanced endosomal escape, excellent safety profiles, and rapid clearance.^[^ ^171^ ^]^ Moderna mainly focuses on the development of lipid‐delivered personalized cancer mRNA vaccines. Identification and selection of appropriate immunogenic neoantigens are critically important for the effectiveness of cancer mRNA vaccines. Moderna has developed different pipelines to identify neoantigens that can be recognized by intratumor T cells, such as discovering driver mutations by exome sequencing of tumor samples in the development of product mRNA‐4650. Moderna also has TAA encoded mRNA vaccine products under clinical development, such as mRNA‐5671 for the treatment of patients with Kirsten rat sarcoma viral oncogene homolog (KRAS) mutant advanced cancers (NCT03948763, Table 2). The recent remarkable success of mRNA‐lipid in the deployment of COVID‐19 mRNA vaccines is fostering the research interest and clinical development of mRNA therapeutics delivered by nanoformulations in cancer immunotherapy.^[^ ^172^, ^173^, ^174^ ^]^ The efficiency of nanoformulations targeting immune cells relies on the chemophysical features of nanoformulations, administration routes, and subsets of immune cells. For example, near neutral lipid‐based mRNA nanoformulations were found efficiently targeting splenic APCs post intravenous administration, demonstrating potent cancer immunotherapy in preclinical and clinical studies.^[^ ^167^, ^168^ ^]^ Following intravenous administration, the reduction responsive nanogel was found efficiently targeting activated T cells for elimination of established tumors,^[^ ^85^ ^]^ which supported its movement from bench to clinical trials.

## 4 Conclusions and Future Directions

**Figure 14:**
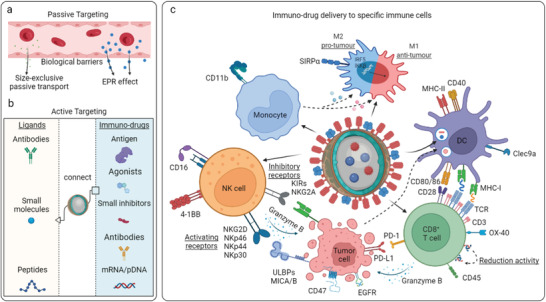
Illustrated are current advanced nanoformulations a) passively and b) actively targeting delivery of immuno‐drugs to tissue‐specific immune cells, and c) the associated mechanisms by which nanoformulations regulate the functions of each specific subset of immune cells for enhanced cancer immunotherapy. ‐Created with Biorender.com.

The use of nanomaterials for targeted chemotherapy has been approved in clinical practice, which enables selective tumor accumulation of systemically administrated cytotoxic chemotherapeutics. Modulation of the nanomaterial constructs, such as small sizes and surface decoration with targeting ligands, improves tumor‐specific targeting and consequently reduces off‐target adverse effects. Recent research interest in nanomaterial delivery platforms has been shifting rapidly toward cancer immunotherapy for improved implementation of therapeutic immuno‐drugs. Current summary reports mainly focus on the targeting strategies (both local and systemic administration) for the modulation of tumor specific immune cells, structural cells, and physiology features in TME.^[^ ^4^, ^175^, ^176^ ^]^ Local administration offers an easy targeting approach, but not practical in the case of hard‐to‐reach cancer sites. Besides, establishing successful anti‐tumor immunity requires coordination of innate and adaptive immune system across tissues, involving lymphoid organs, blood stream, or tumor tissues. In view of these two points, this review highlights recent advances in systemically administrated immuno‐drug nanoformulations (**Figure** **14**) that are capable to target specific subsets of immune cells located in lymphoid organs, bloodstream, and tumor sites for enhanced cancer immunotherapy. The directing strategies of nanoformulations consist of passive targeting (Figure 14a) driven by size exclusive transport or EPR effect and active targeting (Figure 14b) driven by the conjugated specific ligands, such as mAbs, peptides, and small molecules. The exemplified nanoformulations were engineered to direct immuno‐drugs to specific subsets of DCs, T cells, NK cells, TAMs, or monocytes, to promote their functional activity or restore impaired function, thereby enhancing anti‐tumor immunity (Figure 14c). The associated mechanistic insights into the regulation of anti‐tumor immunity are summarized in Table 1. Immune cell‐targeting nanoformulations represent an attractive strategy for cancer therapy, potentiating the efficacy and decreasing off‐target toxicity. Some intriguing candidates are currently under clinical studies (Table 2). Despite the advantages and rapid progress, there are still several challenges and obstacles to be addressed for advancing the clinical translation of these nanoformulations in cancer treatment.

1) Differences in species‐specific immune system: The choice of preclinical settings in the evaluation of therapeutic nanoplatforms is critically important for their translation from animals to humans. For instance, mouse xenograft tumor models are simple to implement, while do not closely replicate the human oncology.^[^ ^177^ ^]^ In addition, the species‐specific immune systems are different in mice and humans,^[^ ^178^, ^179^ ^]^ thus the anti‐tumor immune response in mice hardly reflects those in humans. Patient‐derived xenograft (PDX) model is biologically more relevant to human settings, but the immunodeficient animals require to be humanized with human immune system, such as engraftment of peripheral blood mononuclear cells.^[^ ^177^, ^180^ ^]^ Humanized mouse models serve as a preclinical bridge, improving the understanding of the immune functions of novel immuno‐drug nanoformulations in adoptive T cell therapies as well as therapeutic neoantigen vaccines. Despite some success, humanized mouse models remain constrained by low engraftment rate, long‐term duration, varieties in MHC antigens, and suboptimal development of lymphoid organs. The genetically engineered mouse models that enable spontaneous development of cancer are closely relevant to the features of TME in humans.^[^ ^177^ ^]^ For example, HPV transgenic mice resemble closely the immune signatures of the HPV associated squamous cell carcinoma,^[^ ^181^, ^182^ ^]^ which is an ideal mouse model to investigate the modulation of nanoformulations on tumor infiltrating immune cells. It requires interdisciplinary team engaged in a close and intense collaboration, across the scientists in material science and onco‐immunology and clinicians. Thus, whether the outstanding immuno‐therapeutic efficacy and safety achieved in mice models can predict the performance in human clinical trials remains to be extensively evaluated in the future.
2) Feasibility of scale up production: Cost‐effective and feasible large‐scale manufacturing of these nanoformulations are major concerns that prevent their clinical translation. Usually, new delivery systems that utilize Food and Drug Administration (FDA)‐approved materials (i.e., lipid, polymer) are more likely to move from the lab toward the clinic than those unapproved ones.^[^ ^183^, ^184^ ^]^ As yet, of all the submissions to FDA for drug products using nanoplatforms, liposome was reported to be the most prevalent category, mainly owing to the simplicity in manufacturing at large scale.^[^ ^185^ ^]^ Many FDA‐approved nanoformulation modulated immunotherapy has already been applied in clinic for cancer treatment (Table 2), most of which are lipid‐based formulations. For example, the technology of using lipids to deliver mRNA to DCs previously reported in Nature^[^ ^167^ ^]^ is being tested in clinical trials in patients with melanoma (NCT02410733). However, in the context of nanoplatforms for T‐cell targeted therapy, many studies involved complex design process. With the formulation complexity increasing, manufacturing costs and the regulatory procedures to prove safety and effectiveness increase as well.^[^ ^186^ ^]^ Thus, in future studies, researchers in this field should keep the ultimate goal of the nanomedicine‐ “widespread clinical application” in mind, when designing the nanomaterial solutions.
3) Difficulties in precise targeting to tissue‐specific subsets of immune cells: It remains a challenge to completely resolve off‐target immune toxicities while maintaining sustained anti‐tumor effects in patients receiving immuno‐drugs. Nanoparticles should be engineered to precisely target and modulate tissue‐specific immune cells, reducing adverse effects. The accumulated evidence on ligands revealed that distinct subsets of immune cells can express the same biomarkers. For instance, M1‐ and M2‐ phenotypes of TAMs display shared mannose/galactose receptors.^[^ ^187^ ^]^ Similar to T cells, NK cells express several immune checkpoint molecules on their surface (e.g., TIM‐3, CTLA‐4, and PD‐1),^[^ ^188^, ^189^, ^190^ ^]^ along with some intracellular signaling components (i.e., CDk8, Cbl‐b).^[^ ^191^ ^]^ Thus, nanoformulations targeting these shared components can stimulate undesired immune cell responses in cancer treatment, which might inevitably increase unwanted side effects. To advance nanoformulations targeting specific receptors, receptor humanized mouse models are desired to evaluate the efficacy and safety.^[^ ^191^ ^]^ Also, cancer patients often receive substantial conventional therapies (e.g., radiotherapy and chemotherapy) prior to immunotherapy. Thus, the influence of radio/chemo‐therapeutics should be explored on the biomarkers expressed on patient immune cells for the selection of accurate immune cell‐targeted therapies.
4) Exploration of new mechanisms at the interface of nanoformulations and immune system: The rapid development of cutting‐edge biotechnologies, such as spatial transcriptomics, scRNA sequencing, and CITE‐seq that enables gaining information of surface proteins using available antibody‐bound oligos in RNA sequencing, have advanced our understanding on the cross‐talk between structure cells and immune cells either at normal^[^ ^192^ ^]^ or cancerous tissues.^[^ ^193^ ^]^ The integration of multiple high dimensional multiomics analysis approaches enables identification of new subtypes of disease or tissue specific immune cells and their functions on immune regulation as well as receptor‐ligand networks, deepening our knowledge of immune modulation mechanisms across different organs. The applications of these technologies would uncover novel interaction mechanisms occurring at the interface of nanoformulations and tissue resident immune cells at the injection sites, bloodstream, lymphoid organs, and tumor sites. The observations at mRNA levels are not always correlated well with functional protein levels, thus discoveries derived from these high dimensional sequencing data should be validated at protein levels using biomolecular tools and (Symphony) multichannel flow cytometry analysis. The newly defined mechanisms associated with nano‐immunological activities will provide new guidelines for engineering the next generation of nanoformulations.
5) Comprehensive safety evaluation: The potential toxicity of these fascinating nanoparticles is a burning matter, restraining their clinical translation. Aside from nanotoxicity^[^ ^194^ ^]^ associated with low degradation, DNA damage, or metal poisoning on the central nervous system, nano‐immuno formulations could also potentially cause a diverse range of chronic and irreversible immune‐related adverse events.^[^ ^18^ ^]^ Upon entering biological systems, nanoformulations tend to form a corona by interaction with proteins or lipids in the biological fluids.^[^ ^195^ ^]^ Protein corona was discovered responsible for recognition by innate immune cells and triggering adaptive immune response.^[^ ^195^ ^]^ For example, APC can recognize and uptake nanoparticles with a corona formed by serum components via the lipoprotein receptors. The interactions between nanoformulations and biological molecules, tissues, immune cells, and intracellular biomolecules, determine the fate and performance of nanoformulations in vivo. The study of corona‐nanoformulations is helpful for the prediction of immunological response of nanoformulations, therefore diverse methods have been developed to characterize the nanocomplexes. Cryo‐transmission electron microscopy can visualize the network of corona on the surface of nanoparticles.^[^ ^196^ ^]^ Chemical analysis methods can reveal the compositional profiling of corona in combination with mass spectrometry.^[^ ^197^ ^]^ Affinity chromatography is able to quantitatively measure the total protein in corona adsorbed on organic nanoparticles.^[^ ^198^ ^]^ Recently, a biolayer interferometry‐based fishing strategy was developed for in situ analysis of corona protein adsorption and dissociation processes.^[^ ^199^ ^]^ In the context of tracking the distribution of nanoformulations in the immune system, in vivo imaging and inductively coupled plasma analysis in combination with fluorescence‐activated cell sorting technology offer quantitative approaches to analyze the accumulation of nanoformulations in specific subsets of immune cells in lymphoid organs. The activation status of immune cells induced by nanoformulations can be revealed by flow cytometry analysis. The advanced knowledge in the interactions between nanoformulation and proteins or immune cells provides insights into nano‐protein complexes mediated immune response and potential adverse immunotoxicity. For these reasons, the biological safety of engineered nano‐immune formulations should be systematically evaluated from different aspects, though current preliminary data did not show obvious acute toxicities in preclinical and clinical studies.

Current emerging nanoengineering technologies have shown great potential in amplifying the potency and reducing adverse effects of immunotherapeutic substances. Some investigative nanoformulation‐based targeted cancer therapies are currently under clinical studies. Accumulated evidence exists suggesting that the physiochemical features of nanomaterials along with the immuno‐drugs determine the modulation of immune cells, initiating and shaping anti‐tumor immunity. However, the associated mechanisms remain not fully understood. The advances in highly dimensional biological technologies offer a high possibility to discover unexplored mechanisms, broadening and deepening the knowledge in this field. Close collaborations among nano‐engineers, onco‐immunologists, and bioinformaticians are encouraged to foster the translation of nanoformulations, benefiting cancer patients in the future.

## Conflict of Interest

The authors declare no conflict of interest.
